# DIX domain containing 1 (DIXDC1) modulates VEGFR2 level in vasculatures to regulate embryonic and postnatal retina angiogenesis

**DOI:** 10.1186/s12915-022-01240-3

**Published:** 2022-02-10

**Authors:** Yeaji Kim, Dong Young Kim, Haiying Zhang, Cho-Rong Bae, Daehyeon Seong, Yeomyung Kim, Jaewhan Song, Young-Myeong Kim, Young-Guen Kwon

**Affiliations:** 1 Department of Biochemistry, College of Life Science and Biotechnology, Yonsei University, Seoul, Republic of Korea; 2 Present address: Division of Radiation Biomedical Research, Korea Institute of Radiological and Medical Sciences, Seoul, Republic of Korea; 3 R&D Department, Curacle Co. Ltd, Seongnam-si, Republic of Korea; 4 Vascular System Research Center, Kangwon National University, Chuncheon, Republic of Korea

**Keywords:** VEGFR2, Dvl2, Wnt/β-catenin signaling, DIXDC1, Angiogenesis, Neovascularization

## Abstract

**Background:**

In sprouting angiogenesis, VEGFR2 level is regulated via a fine-tuned process involving various signaling pathways. Other than VEGF/VEGFR2 signaling pathway, Wnt/ β-catenin signaling is also important in vascular development. However, the crosstalk between these two signaling pathways is still unknown to date. In this study, we aimed to investigate the role of DIX domain containing 1 (DIXDC1) in vasculature, facilitating the crosstalk between VEGF/VEGFR2 and Wnt/ β-catenin signaling pathways.

**Results:**

In mice, DIXDC1 deficiency delayed angiogenesis at the embryonic stage and suppressed neovascularization at the neonatal stage. DIXDC1 knockdown inhibited VEGF-induced angiogenesis in endothelial cells in vitro by downregulating VEGFR2 expression. DIXDC1 bound Dishevelled Segment Polarity Protein 2 (Dvl2) and polymerized Dvl2 stabilizing VEGFR2 protein via its direct interaction. The complex formation and stability of VEGFR2 was potentiated by Wnt signaling. Moreover, hypoxia elevated DIXDC1 expression and likely modulated both canonical Wnt/β-catenin signaling and VEGFR2 stability in vasculatures. Pathological angiogenesis in DIXDC1 knockout mice was decreased significantly in oxygen-induced retinopathy (OIR) and in wound healing models. These results suggest that DIXDC1 is an important factor in developmental and pathological angiogenesis.

**Conclusion:**

We have identified DIXDC1 as an important factor in early vascular development. These results suggest that DIXDC1 represents a novel regulator of sprouting angiogenesis that links Wnt signaling and VEGFR2 stability and may have a potential role in pathological neovascularization.

**Supplementary Information:**

The online version contains supplementary material available at 10.1186/s12915-022-01240-3.

## Background

Angiogenesis, the formation of new blood vessels from an existing vasculature, plays an essential role in embryogenesis and is associated with the pathogenesis of various human disorders [1–3]. This is a complex process involving endothelial cells (ECs), soluble factors, and extracellular matrix (ECM) [4, 5]. Multiple vascular endothelial growth factors (VEGFs), including VEGF-A, VEGF-B, VEGF-C, and VEGF-D, interact with VEGF receptors such as VEGFR1, VEGFR2, and VEGFR3 [6, 7]. In particular, the binding of VEGF-A to its receptor VEGFR2 plays a major role in pathophysiological neovascularization and its activation mediates diverse angiogenic signaling pathways leading to EC proliferation, migration, lumen formation, and permeability [8, 9].

The role of VEGF-A/VEGFR2 signaling in tip and stalk cell determination of developing vascular trees is well recognized. Endothelial VEGFR2 on the sprouting front receives VEGF-A secreted from avascular areas and regulates the characteristics of tip cells, which show filopodial extension. Increased VEGFR2 signaling in tip cells induces Dll4 expression, and after the binding of Dll4 with Notch positive cells, governs the formation of ‘stalk cells’ that are less motile but support sprouting and vascular extension [6, 10]. Meanwhile, Dll4/Notch signaling suppresses tip cell formation to regulate tip and stalk cell ratio, while Notch gain-of-function results in reduced filopodia and branching [11]. Moreover, Dll4-Notch signaling leads to downregulation of VEGFR2 [12]. Thus, spatial and timely crosstalk between VEGFR2 and Notch pathways is essential for vascular pattern formation [13].

Wnt signaling plays an important role in vascular development and organ-specific EC differentiation [14]. In canonical Wnt signaling, which has been widely studied, the extracellular Wnt ligand Wnt3a regulates the expression of specific target genes by modulating the β-catenin level [14–17]. During angiogenesis, β-catenin binds VE- and N-cadherins in ECs at cell-to-cell adherens junctions and stabilizes their interaction with the cytoskeleton [16] to reinforce cell-to-cell adhesion and tissue integrity [14]. It has recently been reported that endothelial β-catenin signaling fosters angiogenesis in the central nervous system by promoting VEGF/VEGFR2 signaling [18]. Although both VEGF and Wnt signaling are involved in the development of vascular trees, their precise role in sprouting angiogenesis and determining vascular pattern remains elusive.

Three proteins that contain DIX domain- Dvl, DIXDC1, and Axin form the main framework of canonical Wnt signaling [19]. Stimulation by Wnt ligands trigger polymerization of Dvl by DIXDC1, resulting in the activation of downstream signaling [20]. DIXDC1 regulates Wnt signaling via its association with Dvl through the head of Dvl-DIX and the tail of DIXDC1-DIX, resulting in the biologically active state of Dvl2 [19]. DIXDC1 is ubiquitously expressed and extensively studied in various types of cancer and uncontrolled DIXDC1 expression increases tumor size and metastasis and leads to a poor prognosis [21–25]. In addition, previous studies have suggested that DIXDC1 is a positive regulator of neuronal developments [26, 27] and has been involved in recovery of nerve injuries [28, 29]. However, the biological functions of DIXDC1 in the vascular system have not been elucidated. Through gene targeting of *DIXDC1* in mice, we showed that DIXDC1 is a regulator of sprouting angiogenesis and that it modulates VEGFR2 stability in vasculature.

## Results

### DIXDC1 knockout (KO) in mice resulted in retardation of angiogenesis

**Fig. 1:**
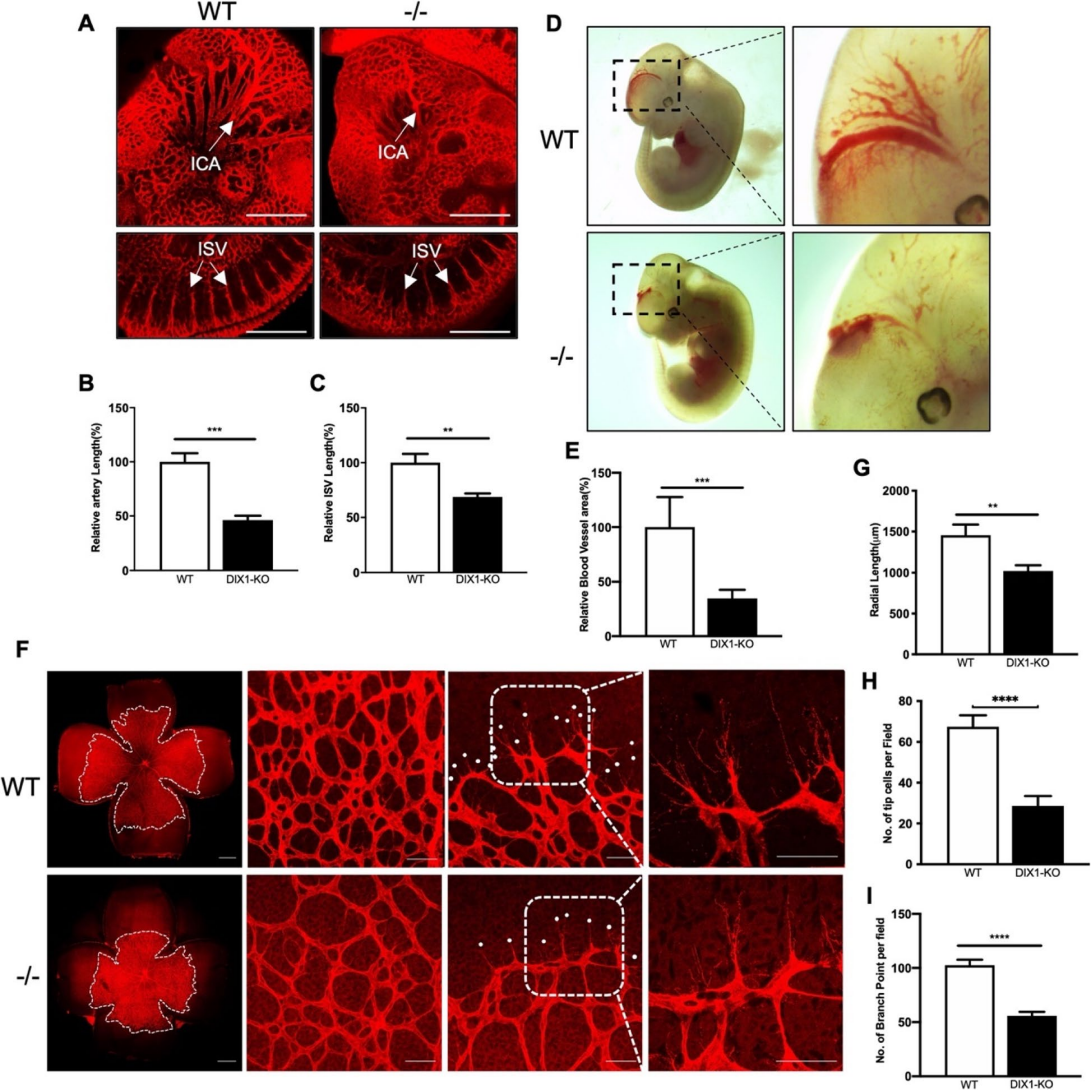
DIXDC1-KO mice showed delayed angiogenesis in embryonic and postnatal stage. **A** Whole-mount preparation of E10.5 embryo from WT and DIXDC1-KO mice immunostained for CD31. Blood vessel density and intersomitic vessel length decreased in DIXDC1-KO embryo compared to the WT. *n* = 5 per group. ICA, internal carotid artery; ISV, intersomitic vessels. Scale bars 500 μm. **B, C** Quantification of A (percentage of control). **D** Lateral view of unfixed E12.5 WT and DIXDC1 knockout embryo. *n* = 5 per group. **E** Quantification of D (percentage of control). **F** Whole-mount preparation of P6.5 retinae from WT and DIXDC1-KO pups immunostained for CD31. Radial length, vascular density, and number of filopodia decreased in DIXDC1-KO mice compared to the WT (individual white dots represent filopodia). *n* = 5 per group. Scale bars 500 μm and 50 μm. **G, H,** and **I** Quantification of radial length, number of tip cells per field, number of branches per field (percentage of control). All experiments were repeated on at least 3 different sets of WT and KO littermates. *p < 0.05, **p < 0.005, and ***p < 0.0001, by paired, 2-tailed Student’s *t* test. Error bars represent the mean ± SD. Individual values can be found in Additional file 6: Fig. 1

To examine the role of DIXDC1 in vascular development, we analyzed the vascular phenotypes of DIXDC1-KO mice embryo and postnatal retinae. As observed on embryonic day 10.5, internal carotid artery development was delayed in the DIXDC1-KO embryo and the lengths of intersomatic vessels were significantly decreased (Fig. 1A–C). Furthermore, on embryonic day 12.5, lengths of the blood vessels in the midbrain were significantly decreased in DIXDC1-KO embryos compared to those in wild type (WT) embryos (Fig. 1D, E).

We further investigated the role of DIXDC1 expression in developing retinae. Development of superficial plexus of retina was observed using the WT and DIXDC1-KO mice. Immunostaining of the WT and DIXDC1-KO mice retinae on postnatal day 6.5 with anti-CD31 antibodies revealed significant decrease in radial length, blood vessel density, and number of filopodia in the DIXDC1-KO mice (Fig. 1F–I). Since vascular development in the DIXDC1-KO mice is markedly retarded, we examined the fertility and body weights of the DIXDC1-KO mice. DIXDC1-KO mice genotypes were confirmed with the conventional PCR method (Additional File 1: Fig S 1. A, B). The DIXDC1-KO mice were born at the expected Mendelian ratio (Additional File 1: Fig S 1. C) and fertility of the DIXDC1-KO mice did not differ from that of the WT mice (Additional File 1: Fig S 1. D). In contrast, the body weights and sizes of the DIXDC1-KO mice were smaller than those of the WT mice by up to 4 weeks (Additional File 1: Fig S 1. E, F), suggesting delayed embryonic development in the DIXDC1-KO mice. These results suggest that DIXDC1 is an important factor in developmental angiogenesis, both at the embryonic and early postnatal stages.

### DIXDC1-KO mice have reduced angiogenic ability

**Fig. 2:**
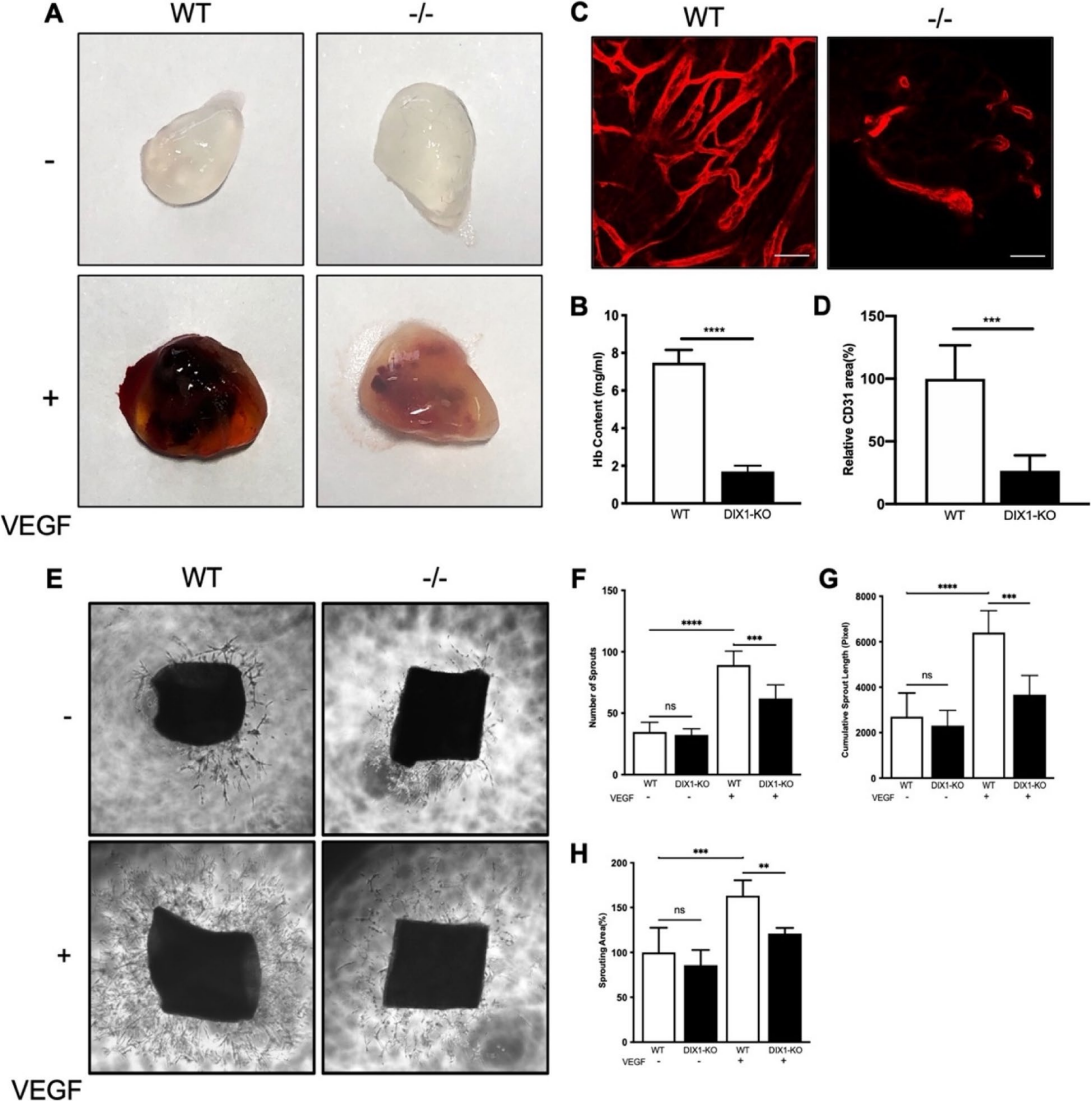
DIXDC1-KO mice had attenuated angiogenic potential. **A** Matrigel plug implanted into 8-week-old male WT and DIXDC1-KO mice for 7 days (murine VEGF-A 50 ng). *n* = 5 per group. **B** Quantification of hemoglobin(mg/ml) extracted from Matrigel plugs from WT and DIXDC1-KO mice. *n* = 5 per group. **C** CD31 immunostaining of Matrigel plugs implanted into 8-week-old WT and DIXDC1-KO mice for 7 days. Scale bars 50 μm. **D** Quantification of CD31 immunostaining of Matrigel plugs. **E** Murine aortic ring assay of 8-week-old WT and DIXDC1-KO mice (murine VEGF-A 50 ng). *n* = 5 per group. **F, G,** and **H** Quantification of number of sprouts, cumulative sprout length (in pixel), and sprouting area (percentage of control). All experiments were repeated on at least 3 different sets of WT and KO littermates. *p < 0.05, **p < 0.005, and ***p < 0.0001, by paired, 2-tailed Student’s *t* test and one-way ANOVA. Error bars represent the mean ± SD. Individual values can be found in Additional file 6: Fig 2

To further determine the angiogenic ability of the DIXDC1-KO mice, 8-week-old WT and DIXDC1-KO male mice were subjected to Matrigel plug assay. The infiltration of hemoglobin (Fig. 2A, B) and ECs (Fig. 2C, D) were significantly decreased in a VEGF-A-dependent manner in the DIXDC1-KO mice compared to those in the WT mice. Interestingly, infiltration of hemoglobin and ECs did not occur in the groups without VEGF-A treatment. Aortic ring assay showed that the sprouting ability of the aorta was decreased in the DIXDC1-KO mice compared to that in the WT mice regardless of VEGF-A treatment (Fig. 2E–H). These results suggested that DIXDC1 might be involved in VEGF-induced sprouting of EC. Reduced EC infiltration and sprouting ability in the DIXDC1-KO mice suggest that DIXDC1 is an important factor in sprouting angiogenesis.

### DIXDC1 regulates angiogenic ability of ECs

**Fig. 3:**
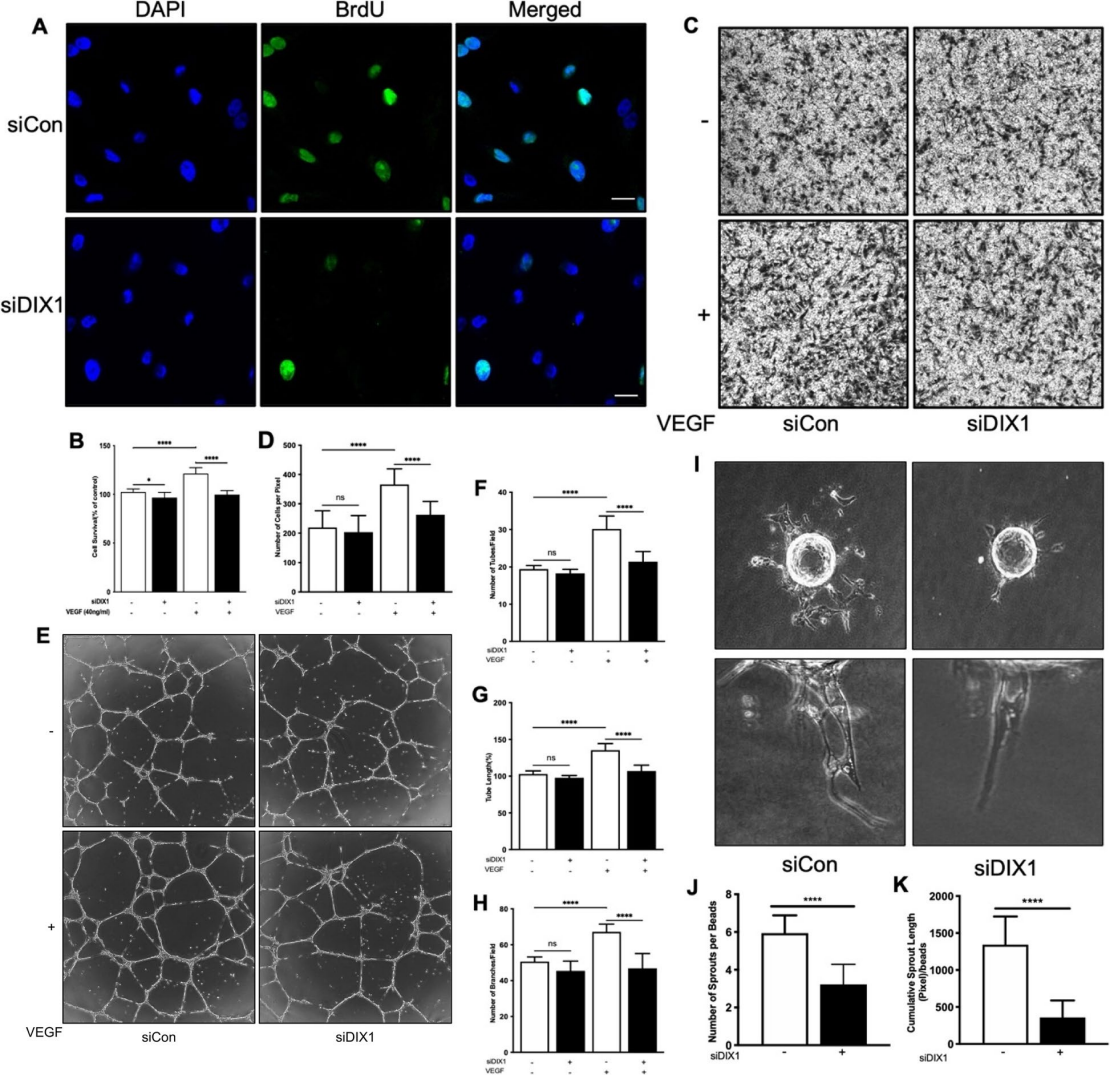
DIXDC1 knockdown in EC result in reduced angiogenic potential. **A** BrdU immunostaining of EC treated with either control siRNA or DIXDC1 siRNA (60 nM). Scale bars 50 μm. **B** MTT assay of EC treated with control siRNA or DIXDC1 siRNA in basal and VEGF-A-treated condition (percentage of control). **C** Transwell migration assay of EC after silencing of DIXDC in EC. Migration of EC was significantly decreased when DIXDC1 is silenced in both basal and VEGF-A-treated condition (40 ng/ml). **D** Quantification of transwell migration assay. **E** Ability of tube-like structure formation of EC decreased when expression of DIXDC1 was suppressed in EC in both basal and presence of VEGF-A. **F, G,** and **H** Quantification of tube formation assay (Percentage of control). **I** Sprouting of EC was decreased when DIXDC1 expression was knocked down in EC. **J, K** Quantification of sprouting assay. All experiments were repeated at least 5 different sets of ECs treated with siRNA or DIXDC1 siRNA. *p < 0.05, **p < 0.005, and ***p < 0.0001, by one-way ANOVA and 2-tailed Student’s *t* test. Error bars represent the mean ± SD. Individual values can be found in Additional file 6: Fig. 3

Since the DIXDC1-KO mice exhibited reduced angiogenic ability, we analyzed the angiogenic ability of ECs. Affymetrix gene chip analysis [30] revealed that DIXDC1 expression in ECs was higher in the outgrowth endothelial (OEC) stage than in the human umbilical cord blood-derived mononuclear cell (UCB-MNC) stage (Additional File 2: Fig. S 2 A), and this was confirmed with RT-qPCR (Additional File 2: Fig. S 2 B). It has been previously reported that murine Dixdc1 promotes G1-S phase transition [28]. Therefore, 5-bromo-2'-deoxyuridine (BrdU) assay was used to measure cell cycle progression. To perform in vitro experiments, the optimal DIXDC1 siRNA treatment condition was determined (Additional File 2: Fig. S 2 C-I). DIXDC1 knockdown in ECs decreased the incorporation of BrdU into DNA, indicating that DIXDC1 regulates cell cycle in these cells (Fig. 3A). Since delayed cell cycle in ECs may decrease cell proliferation, we performed MTT assay of basal and VEGF-A-treated cells. Proliferation was decreased in DIXDC1 knockdown ECs compared to that in the control cells in the VEGF-A treated group (Fig. 3B). Migration assay of basal and VEGF-A-treated cells in a transwell chamber revealed that the chemotactic mobility of DIXDC1 knockdown ECs was decreased in the presence of VEGF-A (Fig. 3C, D).

Further, tube formation assay showed that tube-like structure formation was decreased in the VEGF-A treated DIXDC1 knockdown ECs (Fig. 3E–H). Moreover, in vitro sprouting assay that was used to recapitulate the key stages of angiogenesis revealed significant decrease in sprouting of DIXDC1 knockdown ECs (Fig. 3I, J, and K). These results indicate that DIXDC1 is a significant factor in VEGF-induced angiogenesis in ECs.

### DIXDC1 regulates the basal VEGFR2 level

**Fig. 4:**
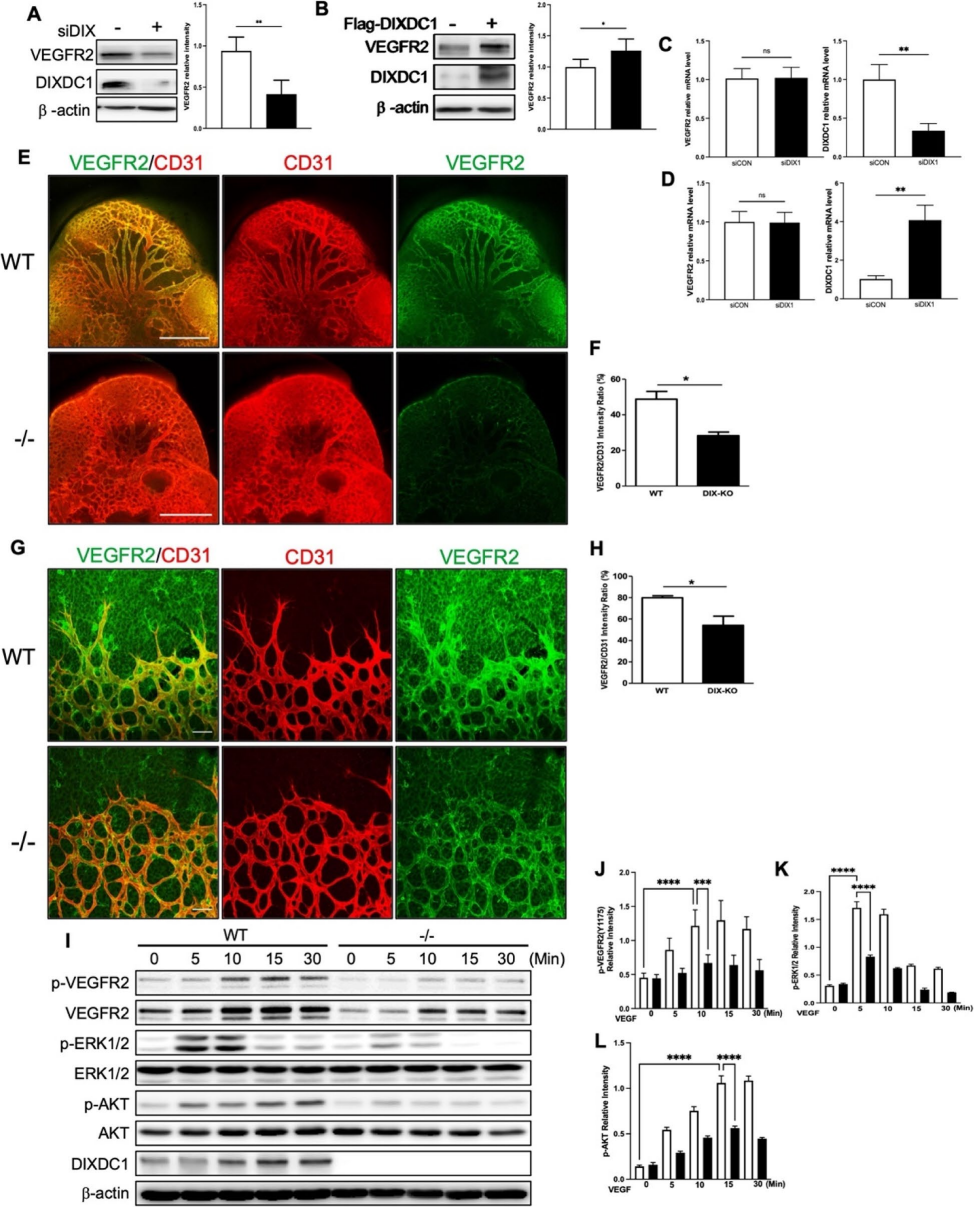
DIXDC1 regulates VEGFR2 level in vitro and in vivo. **A** VEGFR2 protein expression level was reduced when DIXDC1 was suppressed in EC by using DIXDC1 siRNA (60 nM). **B** Significantly increased VEGFR2 protein expression was observed when DIXDC1 was overexpressed in EC. **C**, **D** Relative mRNA level of VEGFR2 and DIXDC1. **E** Whole-mount staining of E10.5 WT and DIXDC1 KO embryos with CD31 and VEGFR2, demonstrating overall decrease in VEGFR2. *n* = 5 per group. Scale bars 500 μm. **F** Quantification of **E** (percentage of control). **G** Whole-mount staining of retinae of P8.5 retinae from WT and DIXDC1-KO pups showing decreased VEGFR2 expression. *n =* 6 per group. Scale bars 500 μm. **H** Quantification of **G** (percentage of control). **I** MLEC from WT and Dixdc1 KO mice at p9.5 displaying reduced VEGFR2 level leading to decreased downstream VEGFR2 signaling. **J, K,** and **L** Quantification of relative intensity of p-VEGFR2, p-AKT, and p-ERK1/2 of MLEC western blot. All experiments were repeated at least 5 different sets of ECs treated with siRNA or DIXDC1 siRNA. All experiments were repeated at least 3 different sets of WT and DIXDC1-KO littermates. *p < 0.05, **p < 0.005, and ***p < 0.0001, by paired, 2-tailed Student’s *t* test and one-way ANOVA. Error bars represent the mean ± SD. Individual values can be found in Additional file 6: Fig 4

Reduction of VEGF-A-induced angiogenesis in DIXDC1 knockdown ECs suggests that DIXDC1 may affect the level of VEGFR2. DIXDC1 knockdown in ECs decreased VEGFR2 expression at the protein level but did not affect the mRNA level (Fig. 4A, C). DIXDC1 overexpression in ECs influenced the basal level of VEGFR2 in these cells; DIXDC1 overexpression in ECs increased the basal level of VEGFR2 but did not affect the mRNA level (Fig. 4B, D). These results suggest that DIXDC1 may elevate post-translational VEGFR2 level.

*Dixdc1* knockdown in mice resulted in the retardation of developmental angiogenesis, suggesting that Dixdc1 regulates Vegfr2 level in vivo. Embryos of the WT and DIXDC1-KO mice on embryonic day 10.5 were co-immunostained with antibodies against VEGFR2 and CD31. Decreased Vegfr2 expression in the DIXDC1-KO embryos might have delayed the development of the DIXDC1-KO embryos (Fig. 4E, F). Co-immunostaining of retinae from the WT and DIXDC1-KO mice with antibodies against CD31 and VEGFR2 revealed that Vegfr2 level in retinae was significantly decreased, especially at the filopodia (Fig. 4G, H, Additional File 3: Fig.S3). To further assess the VEGFR2 level in ECs in the DIXDC1-KO mice, mouse lung ECs (MLECs) were isolated [31] at postnatal day 6.5. This isolated mouse pulmonary ECs had decreased basal VEGFR2 level and impaired VEGF-induced downstream signaling (Fig. 4I–L). These results suggest that DIXDC1 is a novel regulator of VEGFR2 that stimulates sprouting angiogenesis by upregulating VEGFR2 level in tip cells.

### DIXDC1 upregulates VEGFR2 via binding with Dvl2

**Fig. 5:**
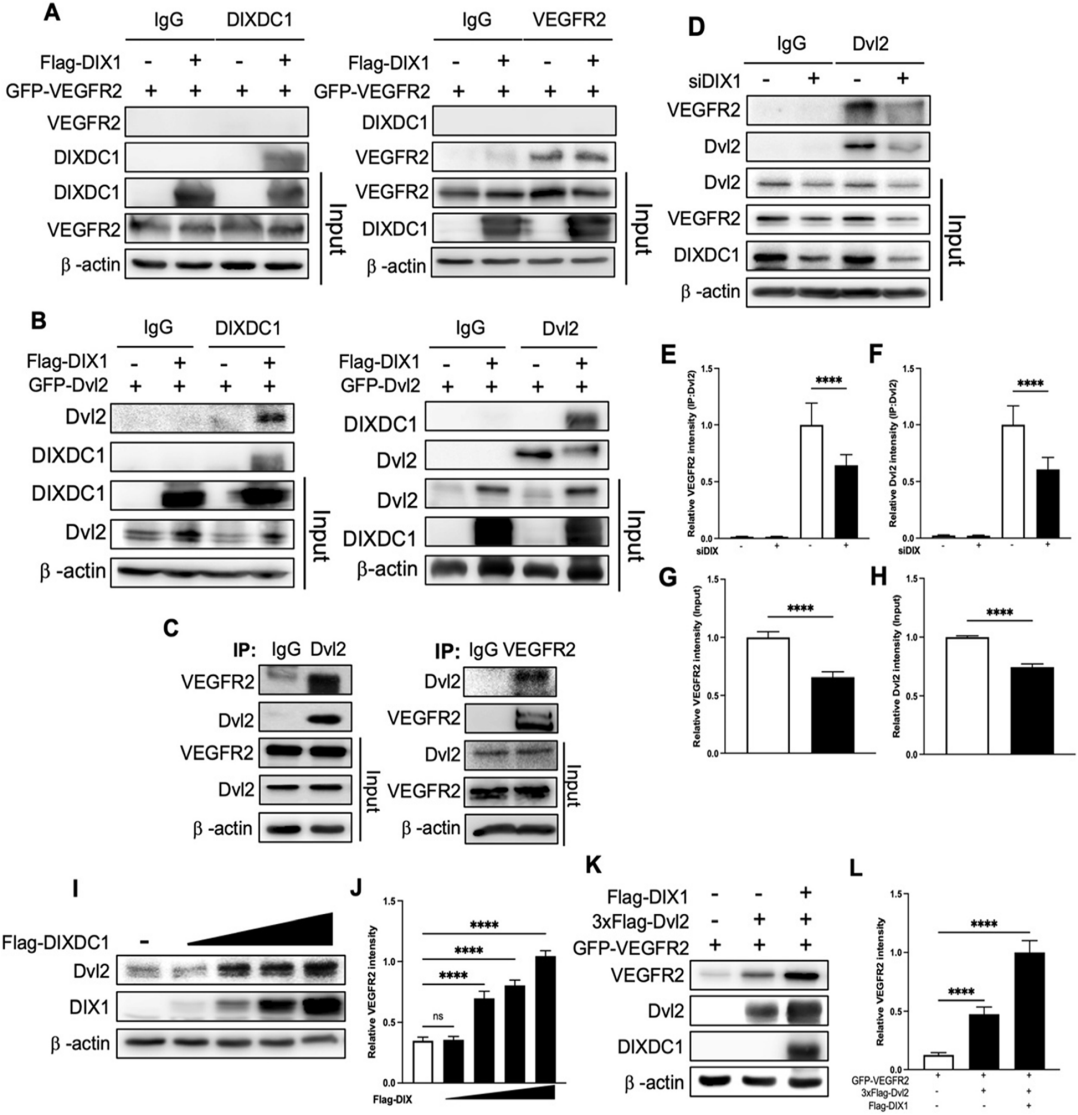
DIXDC1 upregulates VEGFR2 via Dvl2. **A** DIXDC1 or control vector, and VEGFR2 was co-transfected in HEK293T. Immunoprecipitation with antibody against DIXDC1 and VEGFR2 result revealed that no direct interaction between DIXDC1 and VEGFR2. **B** DIXDC1 or control vector, and Dvl2 was co-transfected in HEK293T. Immunoprecipitation with antibody against DIXDC1 and Dvl2 result revealed that there is an interaction between DIXDC1 and Dvl2. **C** Endogenous immunoprecipitation with antibodies against Dvl2 and VEGFR2 and there is an interaction between the two proteins in HUVEC. **D** Control siRNA or siDIX1 is transfected to HUVEC and subjected to immunoprecipitation antibody against Dvl2. **E, F, G,** and **H** Quantification of relative intensity of immunoprecipitated VEGFR2, Dvl2, and input VEGFR2 and Dvl2 of **D**. **I** DIXDC1 was overexpressed and there was a positive correlation between the expression level of DIXDC1 and Dvl2. **J** Quantification of relative intensity of Dvl2 in **I**. **K** DIXDC1, Dvl2, and VEGFR2 were co-overexpressed in HEK293T and the expression level of VEGFR2 and Dvl2 was observed. **L** Quantification of relative intensity of VEGFR2 in **K**. All Experiments were repeated at least 4 different sets. *p < 0.05, **p < 0.005, and ***p < 0.0001, by paired, 2-tailed Student’s *t* test and one-way ANOVA. Error bars represent the mean ± SD. Individual values can be found in Additional file 6: Fig 5

The mechanism by which DIXDC1 regulates VEGFR2 was examined in detail. Co-transfection and immunoprecipitation of DIXDC1 and VEGFR2 in HEK293T showed no binding between them (Fig. 5A), suggesting that DIXDC1 does not regulate VEGFR2 by direct binding. Previous studies have suggested that DIXDC1 and Dvl2 interact directly to increase Wnt signaling [19] and that Dvl2 has a PDZ domain [32], which interacts with VEGFR2 [33, 34]. Therefore, we hypothesized that DIXDC1 regulates Dvl2 level by direct binding, and Dvl2 binds with VEGFR2 via PDZ domain. Immunoprecipitation showed that DIXDC1 and Dvl2 bind directly (Fig. 5B) and that Dvl2 binds with VEGFR2 (Additional File 4: Fig S 4. A). We confirmed this result by endogenous immunoprecipitation with ECs (Fig. 5C). DIXDC1 knockdown in ECs resulted in decreased interaction between Dvl2 and VEGFR2, which might have resulted from decreased levels of both proteins (Fig. 5D–H).

Our results showed direct binding between DIXDC1 and Dvl2, confirming that DIXDC1 regulated Dvl2. DIXDC1 expression levels varied in transfected HEK293T cells and Dvl2 level directly correlated with the DIXDC1 level (Fig. 5I, J), suggesting that DIXDC1 may upregulate Dvl2, and this increased Dvl2 level might further increase VEGFR2 level via direct binding. To observe the changes in VEGFR2 level due to DIXDC1 and Dvl2, VEGFR2, Dvl2, and DIXDC1 were overexpressed in HEK293T. When VEGFR2 was co-transfected with Dvl2, VEGFR2 expression was increased. VEGFR2 expression was further stimulated when DIXDC1 and Dvl2 were co-overexpressed (Fig. 5K, L). These results suggest that DIXDC1 indirectly increases VEGFR2 level by upregulating Dvl2.

### DIXDC1 stimulates Wnt//β-catenin signaling and increases VEGFR2 stability

**Fig. 6:**
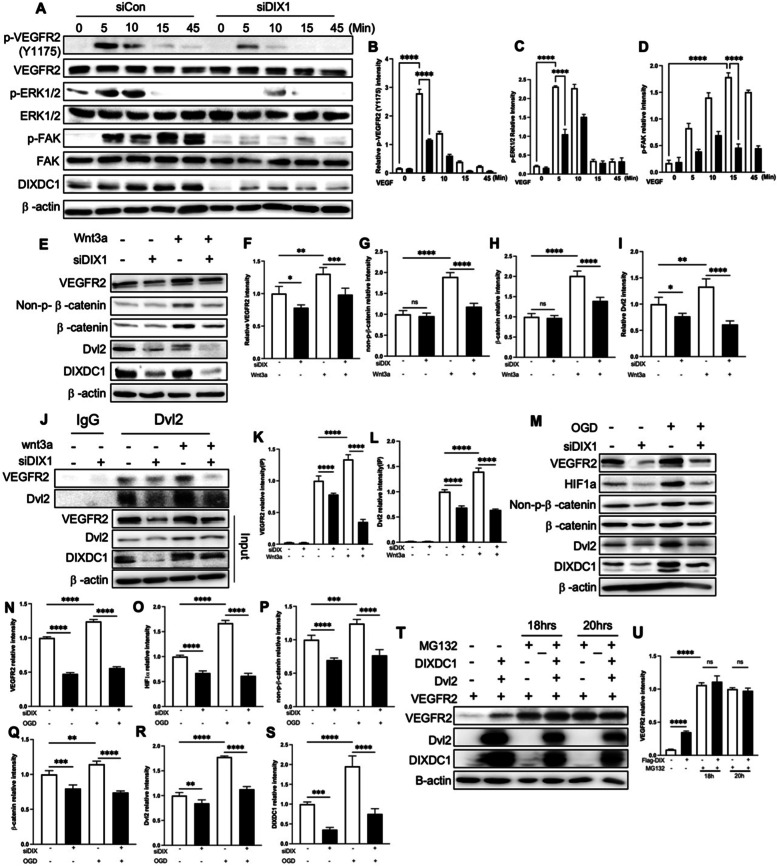
DIXDC1 increases wnt/ β-catenin signaling and VEGFR2 stability via Dvl2. **A** knockdown of DIXDC1 affects the VEGF-induced signaling pathways (40 ng/ml) in HUVEC. **B, C,** and **D** Quantification of relative intensity of p-VEGFR2(Y1175), P-ERK1/2, and p-FAK of **A**. **E** Knockdown of DIXDC1 affects the wnt/β-catenin signaling pathways (200 ng/ml wnt3a) in HUVEC. **F, G, H,** and **I** Quantification of relative intensity of VEGFR2, non-p-β-catenin, β-catenin, and Dvl2 of **E**. **J** Interaction between Dvl2 and VEGFR2 is affected by DIXDC1 and wnt3a (200 ng/ml) in HUVEC. **K, L** Quantification of relative intensity of immunoprecipitated VEGFR2 and Dvl2. **M** Suppression of DIXDC1 affects the level of HIF1a in oxygen glucose-deprived condition in HUVEC. **N, O, P, Q, R,** and **S** Quantification of relative intensity of VEGFR2, HIF1a, non-p-β-catenin, β-catenin, Dvl2, and DIXDC1 of **M**. **T** VEGFR2, Dvl2, and DIXDC1 transfected to HEK293T and treated with MG132. Accumulation of VEGFR2 level was observed with MG132. **U** Quantification of relative intensity of VEGFR2 of **T**. All Experiments were repeated at least 4 different sets. *p < 0.05, **p < 0.005, and ***p < 0.0001, by one-way ANOVA. Error bars represent the mean ± SD. Individual values can be found in Additional file 6: Fig. 6

After demonstrating that DIXDC1 increased VEGFR2 level, we investigated the downstream signaling that ensues VEGFR2, Dvl2, and DIXDC1 overexpression in HEK293T cells. VEGFR2 expression increased with increase in Dvl2 expression and further increased from the combined effects of DIXDC1 and Dvl2. Moreover, downstream signaling (p-AKT and p-ERK) increased as VEGFR2 expression increased (Additional File 4: Fig S 4. B-E). DIXDC1 was knocked down in ECs and VEGF-induced downstream signaling was observed. There was a significant decrease in VEGF/VEGFR2 downstream signaling pathway (Fig. 6A–D). These results indicated that DIXDC1 upregulates Dvl2 and further increases basal VEGFR2 level, stimulating the downstream signaling pathway. Therefore, the effects of the Wnt/β-catenin signaling ligand Wnt3a on VEGFR2, Dvl2, and β-catenin levels were examined in DIXDC1 knockdown ECs. β-catenin accumulation was significantly decreased by reduction in DIXDC1 level and Wnt3a stimulated VEGFR2 expression (Fig. 6E–I). Furthermore, combination of Wnt3a and VEGF-A stimulation increases VEGFR2 further (Additional File 5: Fig S 5 A-E). VEGFR2 level is depended on DIXDC1 level but did not increase VEGFR2 level with suppressed Dvl2 level (Additional File 5: Fig S 5 F G). Increased VEGFR2 expression due to Wnt3a stimulation suggested that the interaction between VEGFR2 and Dvl2 might have resulted from increased binding between the two proteins. Immunoprecipitation of DIXDC1 knockdown ECs stimulated with Wnt3a revealed increased Dvl2 and VEGFR2 interaction (Fig. 6J–L). A previous study has indicated that oxygen deprivation increases both Wnt/β-catenin [35] and VEGF-A/VEGFR2 [36] signaling pathways, and our result demonstrated that DIXDC1 regulates both signaling pathways. DIXDC1 knockdown ECs were investigated under oxygen and glucose deprivation (OGD) condition, and the results indicated significant upregulation of DIXDC1. Consequently, VEGFR2 and Dvl2 expression were also upregulated (Fig. 6M–S). Thus, the upregulation of Dvl2 by DIXDC1 stimulated Wnt and VEGFR2 levels.

DIXDC1 knockdown in ECs resulted in reduced VEGFR2 level in a post-translational manner, likely due to the reduced Dvl2 level that might have affected the stability of VEGFR2. A proteasome inhibitor, MG132, was administered to HEK293T cells transfected with VEGFR2, Dvl2, and DIXDC1. VEGFR2 level was increased by Dvl2 and DIXDC1 overexpression, and VEGFR2 level increased when MG132 was treated (Fig. 6T, U). Thus, DIXDC1 may upregulate Wnt signaling and increase the stability of VEGFR2 via Dvl2.

### DIXDC1-KO mice exhibited decreased neovascularization

**Fig. 7:**
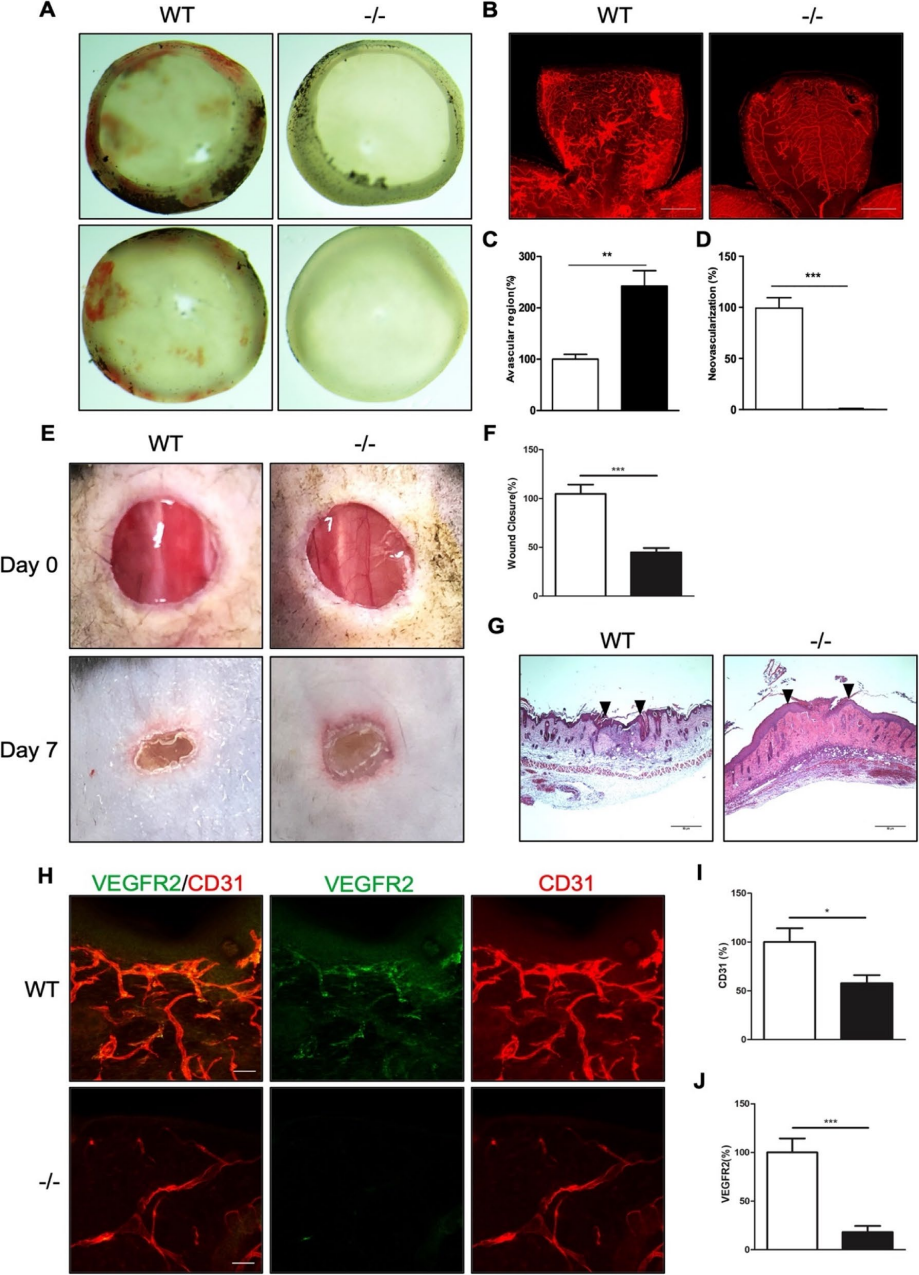
DIXDC1-KO mice displayed decreased neovascularization. **A** Hemorrhage in WT and Dixdc1 knockout mice retinae after exposure to high oxygen level (75% O_2_) for 5 days. (OIR model) *n =* 6 per group. **B** Whole-mount CD31 immunostaining of retinae from **A**. Scale bars 500 μm. **C, D** Quantification of relative avascular region and neovascularization. **E** In vivo wound healing assay of 8-week-old WT and DIXDC1-KO mice. *n =* 6 per group. **F** Quantification of wound healing assay. **G** H&E staining of horizontal section of wound area of WT and DIXDC1-KO mice. Scale bar 50 μm. **H** CD31 and VEGFR2 immunostaining of horizontal section of wounded skin of WT and Dixdc1 KO mice. **I, J** Quantification of relative CD31 and VEGFR2 level. All Experiments were repeated at least 3 different sets of WT and DIXDC1-KO littermates. *p < 0.05, **p < 0.005, and ***p < 0.0001, by paired, 2-tailed Student’s *t* test. Error bars represent the mean ± SD. Individual values can be found in Additional file 6: Fig. 7

Decrease in DIXDC1 level reduced the key signaling pathways that are essential to angiogenesis, and this likely affected neovascularization under pathological conditions. We used an oxygen-induced retinopathy (OIR) model to investigate pathological angiogenesis in the DIXDC1-KO mice. Unfixed retinae showed severe hemorrhage in the WT mice but not in the DIXDC1 KO mice (Fig. 7A). Whole-mount immunostaining of CD31 showed a significant increase in the avascular region, and neovascularization did not occur in the retinae of the DIXDC1 KO mice (Fig. 7B–D). To further confirm reduced neovascularization in the DIXDC1-KO mice, in vivo wound healing assay was used. Wound closure in the DIXDC1-KO mice was significantly reduced (Fig. 7E F), and hematoxylin and eosin (H&E) staining confirmed this (Fig. 7G). Wounded skins in the WT and DIXDC1 KO mice were immunostained with antibodies against CD31 and VEGFR2. CD31 coverage and VEGFR2 expression around the wounded area decreased in the DIXDC1 KO mice compared to that in the WT mice (Fig. 7H–J). Both the OIR model and in vivo wound healing assay indicated that the DIXDC1-KO mice had reduced neovascularization.

## Discussion

We identified DIXDC1 as a novel regulator of sprouting angiogenesis and pathological neovascularization. The DIXDC1-KO mice showed delayed developmental angiogenesis in both embryonic and postnatal stages and showed reduced neovascularization. DIXDC1 knockdown in ECs decreased the angiogenic potential, which was correlated with the phenotypes of the DIXDC1 knockout mice. These phenotypes result from the significant decrease in VEGFR2 level due to its reduced stability. DIXDC1 binds Dvl2, and the polymerized Dvl2 in turn stabilizes VEGFR2 via direct interaction. These findings strongly suggest that DIXDC1 is an important regulator of angiogenesis that modulates Wnt signaling, which increases the stability of VEGFR2 in sprouting tip cells.

Our findings suggest that DIXDC1 is a significant factor in regulating VEGFR2 level in tip cell filopodia. Phenotypes of the DIXDC1-KO mice and DIXDC1 KO ECs were correlated in terms of angiogenic potential. DIXDC1 deficiency significantly decreased sprouting angiogenesis by reducing the number of tip cells in the DIXDC1-KO mice and sprouting of ECs due to decreased level of VEGFR2. VEGF-A/VEGFR2 signaling, which guides filopodial extension from specialized endothelial cells, is essential for the development of postnatal retina [10]. VEGFR2 is prominently expressed on tip cell filopodia in developing retina and detects extracellular VEGF-A gradients [4, 7, 10]. Administration of VEGF-A or VEGFR2-blocking antibodies from postnatal day 4 to 7 effectively reduced EC sprouting and vessel density in the retina [37]. The vasculature of the DIXDC1-KO mice is similar to the phenotype of mice with VEGFR2 blockage in the retina—resulting in significant reduction in EC sprouting and vessel density. Further, we found that DIXDC1 expression in developing retina peaked on postnatal days 5 to 8, which is the period when sprouting angiogenesis occurs rapidly at the superficial plexus. Delay in superficial vascular plexus extension and decreased neovascularization are likely caused by decreased VEGFR2 level in the DIXDC1-KO mice. Phenotypes of the DIXDC1-KO mice and the reduced angiogenic ability of DIXDC1 knockdown ECs suggest a novel VEGFR2 regulatory axis of DIXDC1. Therefore, we hypothesized that DIXDC1 upregulated VEGFR2 in vasculature, which likely increased the sensitivity to VEGF-A and increased sprouting angiogenesis in postnatal retina.

We have shown that the knockdown of DIXDC1 in ECs decreased the accumulation of β-catenin after stimulation by the Wnt ligand Wnt3a. DIXDC1 is a positive regulator of Wnt/β-catenin signaling that upregulates Wnt/β-catenin signaling by converting Dvl2 into its biologically active state [19]. Biologically active Dvl2 forms signalsomes with the Wnt co-receptors LRP 5/6 and Frizzled [38]. Previous studies have suggested that Wnt/β-catenin signaling is an important factor in sprouting angiogenesis. Impaired β-catenin accumulation in ECs resulted in reduced postnatal retinal and brain angiogenesis, with decreased endothelial tip cell formation and sprouting [18]. Furthermore, β-catenin-deficient ECs are less likely to be in a tip cell position while ECs with upregulated β-catenin are likely to be so. Other studies have demonstrated that the blockage of Wnt signaling in ECs reduced the VEGFR2 level [39], which resulted in phenotypes that are similar to those observed in DIXDC1 knockdown ECs. Mutation study on Ctnnb^i∆EC^ showed diminished EC sprouting [40], suggesting that DIXDC1 may regulate the accumulation of β-catenin and affect the sprouting of ECs. Interestingly, we found that the role of DIXDC1 in sprouting angiogenesis was Wnt/β-catenin independent. Stimulation with the Wnt ligand Wnt3a increases VEGFR2 and Dvl2 binding, resulting in increased level of the receptor. Increased VEGFR2 level may stimulate the tip cells with its ligand VEGF-A, and a previous study demonstrated that Wnt/ β-catenin signaling increases VEGF-A level [41]. Thus, DIXDC1 may strengthen the autocrine loop of VEGF-A/VEGFR2 signaling by upregulating VEGF-A via enhanced Wnt/β-catenin signaling and increased stability of VEGFR2 at the tip cells.

Our results demonstrated that DIXDC1 may mediate a crosstalk between two signaling molecules in sprouting angiogenesis by regulating VEGFR2 level and Wnt/β-catenin signaling. We hypothesize that DIXDC1 is an important factor in sprouting ECs that regulates the crosstalk between the two signaling pathways by modulating Wnt/β-catenin signaling, ultimately regulating the level of VEGFR2.

We have suggested the possibility of DIXDC1-mediated regulation of VEGFR2 stability via Dvl2. We found that the interaction between Dvl2 and VEGFR2 and the absence of DIXDC1 and Dvl2 resulted in increased ubiquitination of VEGFR2. Dvl2 is recruited and forms signalsomes with Frizzed and LRP5/6 and prevents the destruction of β-catenin [42, 43]. Dvl2 has three domains, including a central PDZ domain [32], and several studies have indicated the importance of PDZ domain in the stability of VEGFR2. Neuropilin-1 is a VEGFR2 co-receptor with a PDZ domain, and deletion of PDZ domain results in decreased VEGFR2 level [33]. Another protein with a PDZ domain, Syntenin, acts as a scaffold protein to stabilize VEGFR2, and its knockdown reduces VEGFR2 level and shows similar phenotypes as DIXDC1 knockdown ECs [34]. Absence of DIXDC1 and Dvl2 may result in increased proteasomal degradation of VEGFR2, reducing its availability. A previous study on the E1 ubiquitin-activating enzyme UBA1 in ECs provided insight into VEGF-A-independent constitutive degradation of VEGFR2 [44]. Ubiquitin-linked pathway regulates the balance between receptor recycling and degradation, affecting VEGF-A stimulated signal transduction and the endothelial response. Consistent with previous studies, our study suggests that DIXDC1 could be a factor that increases the stability of VEGFR2 by upregulating Dvl2. We intend to investigate the detailed mechanism of VEGFR2 regulation by Dvl2 in the future. Although we demonstrated the role of Dvl2 as a factor that increases the stability of VEGFR2, it is unclear whether it exhibits dimeric interaction with Dvl2 and VEGFR2 or a trimeric interaction involving other proteins. This is an area that will be investigated further in our future study.

We demonstrated that the Wnt/β-catenin signaling ligand Wnt3a increases binding between VEGFR2 and Dvl2, resulting in decreased proteasomal degradation of the receptor. Therefore, we hypothesize that DIXDC1 is likely to be a factor that increases the availability of VEGFR2 and impacts VEGF-A-induced response, affecting its ability to sprout angiogenesis.

This study showed that DIXDC1 is upregulated in hypoxic conditions, resulting in increased Wnt signaling that further increases VEGFR2 level. HIF1α is a major factor in age-related macular degeneration (AMD) [45], diabetic retinopathy [46], and retinoblastoma [47]. Previous studies on OIR have shown upregulation of HIF1α [48], which results in increased neovascularization. Therefore, to better understand the role of DIXDC1 in retina degenerative conditions in humans and support the in vitro finding of our study, we used an OIR model. We demonstrated a reduction of neovascularization and increased avascular region in the DIXDC1-KO mice, which resembles the phenotypes of HIF1α-KO mice. OIR model using conditional HIF1α KO mice demonstrated attenuated level of VEGF, reduced vascular leakage, and alleviated neovascularization in the retina [46]. Consistent with the OIR model, in vivo wound healing in the DIXDC1-KO mice displayed decreased wound closure and recruitment of ECs and VEGFR2. The result of our study suggests that DIXDC1 enhances neovascularization in pathological conditions.

This study also suggests that DIXDC1 is likely to be an inducer of vascular permeability. Previous studies have suggested that VEGFR2 induces vascular permeability via c-Src signaling [49]. VEGF promotes phosphorylation of c-Src at Y418 and modulates adherens junction. It would be an interesting future study to investigate the possible role of DIXDC1 in vascular permeability and related pathological conditions.

Further, this study showed that OGD condition in EC increased β-catenin, VEGFR2, and DIXDC1 levels. This finding is supported by the findings of previous studies on DIXDC1 under hypoxic conditions. Elevated DIXDC1 level was observed after neuronal injury induced by oxygen-glucose deprivation and reoxygenation (OGD/R) [50], and DIXDC1 level was influenced by astrocyte proliferation after a traumatic brain injury [51]. Hypoxia upregulates both Wnt/β-catenin [35, 52] and VEGF/VEGFR2 [36, 53] signaling. In parallel with previous studies, we suggest that HIF1α enhances DIXDC1 level and modulates key signaling pathways under hypoxic conditions.

## Conclusion

In summary, this study demonstrated the importance of DIXDC1 in developmental angiogenesis and neovascularization owing to its role in modulating canonical Wnt/β-catenin signaling, which ultimately regulates the stability of VEGFR2. Our results indicated that DIXDC1 increased Dvl2 via direct interaction, increasing VEGFR2 stability by directly binding with Dvl2. Therefore, we suggest DIXDC1 as a novel regulator of VEGFR2 in vasculature, affecting the ability of ECs in sprouting angiogenesis. Regulation of DIXDC1 is important in both developmental and pathological angiogenesis and targeting DIXDC1 could be a promising approach to the treatment of angiogenesis-related disorders.

## Methods

### DIXDC1 knockout mouse and animal experiments

The DIXDC1 knockout mice (B6.129-Dixdc1^tm1Bnrc^/J, Stock No: 029504) were purchased from The Jackson Laboratory. All mice were maintained on a C57BL/6 background and used between postnatal days 5 to 9 or at 8 weeks of age. Animal experiments were approved by the Yonsei Laboratory Animal Research Center (YLARC) at Yonsei University in Seoul, South Korea. A laminar airflow cabinet was used to maintain all animals under specific pathogen-free conditions. All facilities used for the animal experiments were approved by the Association of Assessment and Accreditation of Laboratory Animal Care, and all the experiments were carried out under the institutional guidelines established for the YLARC at Yonsei University.

### Cell culture

HUVECs were isolated using 250 U/ml Type II Collagenase (Worthington Biochem, Catalog LS004176) in phosphate-buffered saline (PBS) for 10 min at 37 °C, as described previously [54], and cultured on gelatin-coated plates in Medium 199 (HyClone, GE Lifesciences, Catalog SH30253.01) supplemented with 20% fetal bovine serum (FBS), 3 ng/ml basic fibroblast growth factor (bFGF) (R&D Systems), 100 U/ml penicillin-streptomycin (Invitrogen, Thermo Fisher Scientific, Catalog 15140-122), and 5 U/ml heparin. Cells were collected using Trypsin-EDTA (Invitrogen, Thermo Fisher Scientific, Catalog R-001-100) and used for assays up to passage 7. HEK293T was cultured in Dulbecco’s modified Eagle’s medium (DMEM) (HyClone, GE Lifesciences, Catalog SH30243.01) supplemented with 10% FBS and 100 U/ml penicillin-Streptomycin.

### Antibodies and reagents

The following primary antibodies were used: CD31(BD Pharmingen, Catalog 550274); DIXDC1 (Santa Cruz Biotechnology, Catalog sc-377160); VEGFR2 (Cell Signaling Technology, Catalog 9572); Dvl2 (Cell Signaling Technology, Catalog 3216); BrdU (Santa Cruz Biotechnology, Catalog sc-32323); β-catenin (Santa Cruz Biotechnology, Catalog sc-59737); Non-phospho- β-catenin (Cell Signaling Technology, Catalog 19807); DAPI (Sigma-Aldrich, Catalog D9542); β-actin (Thermo Fisher Scientific, Catalog MA5-15739).

The secondary antibodies used were Alexa Fluor 488 (Invitrogen, Thermo Fisher Scientific, Catalog A11006) and Alexa Fluor 594 (Invitrogen, Thermo Fisher Scientific, Catalog A21209).

### RNA interference an RT-qPCR

HUVECs were transfected for 3 h with scrambled siRNA or DIXDC1 siRNA using lipofectamine (Invitrogen, Thermo Fisher Scientific) and assayed 96 h after transfection. The sequence of the DIXDC1 siRNA (5′-GGAAGGAAAUCACCGGUAU-3′) was designed by Dharmacon Inc. (Catalog D-007758-04-0005). For RT-qPCR assays, total RNA (isolated from HUVECs transfected with scrambled siRNAs or DIXDC1 siRNA) was reverse transcribed to cDNA using M-MLV Reverse Transcriptase (Promega, Catalog M170A) and amplified with the primers listed in Additional file 2: Fig. S2D. All RT-qPCR assays were performed at least in triplicate.

### Plasmids

The DIXDC1 Open Reading Frame (ORF)-containing plasmid Flag-DIX was generated. The Dvl2 ORF-containing plasmid 3XFlag-Dvl2(Item #24802) was purchased from Addgene (Cambridge, MA, USA). pEGFP-C2 was used as a transfection control (Clontech).

### Western blot and immunoprecipitation

For the immunoprecipitation of endogenous proteins, HUVECs were cultured as previously described. HUVECs or co-transfected HEK293Ts were lysed in 1 ml NP-40 lysis buffer (50-mM Tris/HCl, pH 8.0, 150-mM NaCl, 1% NP-40, and protease inhibitor cocktail). Cell lysates were centrifuged at 14,000*g* for 15 min at 4 °C. The supernatants were immunoprecipitated overnight with antibodies against Dvl2 (Cell Signaling), VEGFR2 (Cell Signaling), and DIXDC1 (Santa Cruz) at 4 °C, followed by the addition of protein G agarose beads (EMD Millipore) at 4 °C for 3 h. Immunoprecipitates were washed with washing buffer three times and resuspended in SDS-PAGE sample buffer containing β-mercaptoethanol. Samples were further analyzed by western blotting.

### MTT (3-(4,5-dimethylthiazol-2-yl)-2,5-diphenyltetrazolium bromide) assay

HUVECs transfected with scrambled siRNA or DIXDC1 siRNA were seeded on 24-well plates coated with 2% gelatin at a density of 3 × 10^4^ cells/ml. Cells were then starved for 12 h in 1% FBS M199 medium. Then, 40 ng/ml human VEGF-A (Catalog K0921148; Koma Biotech) was treated and 3-(4, 5-dimethyl-2-thiazolyl)-2, 5-diphenyl-2H-tetrazolium bromide (MTT) (0.1 mg/ml) was added after 6 h; cells were incubated at 37 °C for 3 h. Residual MTT was removed, and the crystals were dissolved in dimethyl sulfoxide (DMSO): ethanol (1:1). The absorbance was measured using a spectrophotometer at 560 nm. Each sample was assayed in triplicate, and the assay was repeated three times.

### BrdU assay

HUVECs were transfected with scrambled siRNA or DIXDC1 siRNA and seeded on 35 × 10 mm dishes (Eppendorf AG, Catalog 0030700015) at a density of 6 × 10^5^. M199 media containing 20% FBS and 10 μM BrdU was incubated at 37 °C in a CO_2_ incubator for 24 h. M199 media containing 10 mM BrdU was removed and washed with 1× PBS twice and fixed with 4% paraformaldehyde (PFA) at room temperature (RT) for 10 min. Cells were permeabilized with 0.1% PBST at 4 °C for 5 min and incubated overnight with primary BrdU antibody (Santa Cruz, Catalog sc-32323) at 4 °C with shaking. Cells were washed with PBS and incubated with Alexa Fluor 488 (Invitrogen, Thermo Fisher Scientific) at RT for 1 h with shaking. DAPI was incubated for 15 min and washed with PBS 3 times. Cells were mounted with mounting medium (Dako, Catalog S3023) on cover glasses and photographs were taken with a confocal microscope at a magnification of × 200 (LSM700, Carl Zeiss).

### Migration assay

The chemotactic motility of HUVECs was assayed in Transwell chambers (Corning, Catalog 3470) with polycarbonate filters (8 μm pore size; 6.5 mm diameter). In brief, the bottom end of the filter was coated with 0.1% gelatin. M199 medium (HyClone, GE Lifesciences) containing 1% FBS and 40 ng/ml VEGF-A was added to the lower wells. HUVECs were trypsinized and resuspended in M199 containing 1% FBS to a final concentration of 1 × 10^6^ cells/ml. A 100 μl aliquot of the cell suspension was added to each of the upper wells and incubated at 37 °C for 3 h. Cells were then fixed and stained with H&E. Non-migrated cells on the upper surface of the filter were removed by wiping with a cotton swab, and chemotaxis was quantified by counting the cells that migrated to the lower side of the filter by optical microscopy at magnification of × 200. In total, 10 fields were counted for each assay. Each sample was assayed in triplicate, and the assays were repeated three times.

### In vitro tube formation assay

Tube formation assay was carried out as previously described [55, 56]. Briefly, 250 μl of growth factor–reduced Matrigel Matrix Basement Membrane (Corning, Catalog 354230) was added to a 24-well culture plate (TPP Techno Plastic Products AG, Catalog 92024) and allowed to polymerize for 30 min at 37 °C. Then, 1 × 10^6^ HUVECs transfected with scrambled siRNA or DIXDC1 siRNA were seeded on Matrigel cultures and incubated at 37 °C. Photographs were taken at various time points at a magnification of × 200. The images of the tubes were quantified using ImageJ software (National Institutes of Health).

### In vitro fibrin gel beads assay

In vitro fibrin gel beads assay was carried out as previously described [57]. In brief, HUVECs transfected with either control siRNA or DIXDC1 siRNA were trypsinized and 1 × 10^6^ HUVECs were coated on approximately 2500 Cytodex-3 beads (Amersham Biosciences, Catalog 17-0485-01) overnight at 37 °C. On the following day, the coated beads were washed 3 times with EGM-2 (Lonza, Catalog CC-3162) and 2.0 mg/ml fibrinogen type I (Sigma-Aldrich, Catalog F-8630) solution containing 0.15 U/ml aprotinin (Sigma-Aldrich, Catalog A-1153) at concentration of 500 beads/ml was added. Next, 0.625 U/ml Thrombin (Sigma-Aldrich, Catalog T-3399) was added to a 24-well plate and fibrinogen solution with beads was added to each well by pipetting 4–5 times. After clot formation, 20,000 fibroblasts in EGM-2 were added to each well. Pictures were taken every day for a week with a light microscope at a magnification of × 200. The number and lengths of the sprouts were analyzed using ImageJ.

### Matrigel plug assay

Eight-week-old WT and DIXDC1-KO male mice were injected subcutaneously with 0.6 ml growth factor–reduced Matrigel (Corning) containing 200 ng mouse VEGF-A and 10 U heparin. The injected Matrigel rapidly formed a single, solid gel plug. After 5 days, the animals were sacrificed and the Matrigel plug, which remained intact, was removed. Hemoglobin within the plug was measured using Drabkin’s Reagent (Sigma-Aldrich, Catalog D5941) by comparing with known Hb concentrations. Infiltrated ECs were visualized by immunohistochemistry on cryosectioned slices of the Matrigel plug using anti-CD31 antibody (BD Pharmingen).

### Aortic ring assay

Eight-week-old WT and DIXDC1-KO male mice were used to perform aortic ring assay, as described previously [58]. In brief, aortic rings were isolated from the WT and DIXDC1-KO mice and cut to a fixed size in PBS. Then, 150 μl of growth factor–reduced Matrigel (BD Biosciences) was added to each well of 96-well plates, and the plates were incubated at 37 °C for 10 to 15 min to allow the matrix to polymerize. The aortic rings were then placed on the matrix (1 per well) and fixed onto the polymerized Matrigel by gently pressing the aortic ring into the Matrigel. The plates were incubated further at 37 °C for 10 to 15 min and 100 μl Opti-MEM culture medium (Gibco, Thermo Fisher Scientific) supplemented with 2% FBS and 50 ng/ml murine VEGF-A. The medium was changed on day 2 and then every other day.

### OIR model

The OIR models for the WT and DIXDC1 KO mice were prepared as described previously [59]. Mice at P7 were exposed to a hyperoxic atmosphere (75% O_2_) condition for 5 days and returned to normoxic condition for additional 5 days. The mice were anesthetized and sacrificed using avertin. The eyes were removed and fixed in 4% PFA (pH 7.4) for 30 min at RT. The retinae were isolated and incubated in blocking solution for 1 h (all at RT) and incubated overnight with antibody against CD31 (BD Pharmingen) at 4 °C. After 5 washes in PBS containing 1% Triton X-100, the retinae were incubated with Alexa Fluor 594 (Invitrogen, Thermo Scientific) for 3 h at RT. Retinae were then washed with PBS containing 1% Triton X-100 5 times and postfixated with 4% PFA for 5 min. Retinae were then flat mounted on slides using coverslides and mounting solution (Dako, Catalog S3023) and analyzed using a confocal microscope (LSM 880; Carl Zeiss).

### In vivo wound healing assay

The in vivo wound healing assays for the WT and DIXDC1 KO mice were performed as described previously [60]. In brief, 8-week-old WT and DIXDC1 KO mice were anesthetized using 2.5% avertin and the operative region was created by removing fur with clippers; the skin was wiped with an alcohol swab. Then the wound was created using a sterile 6 mm biopsy punch (Kai Medical, Catalog BP-60F) and circular silicone splint was attached around the wound with the cyanoacrylate adhesive. Circular silicone splint was anchored with 6-0 nylon sutures. Fresh wounds were covered with transparent occlusive dressings and the mice were kept in a laminar airflow cabinet for 7 days. After 7 days, mice were sacrificed, and the diameters of the wounds were measured at the various points. Wounded skin was removed surgically, and the tissues were fixed with 4% paraformaldehyde at 4 °C overnight. The tissues were then transferred to 15% sucrose in PBS and subsequently to 30% sucrose. The wounds were subjected to H&E staining and immunohistochemistry analysis.

### Statistical analysis

Statistics were performed using GraphPad Prism software. Data are presented as means ± SD and analyzed using paired Student’s *t* test or the one-way ANOVA. Differences were considered significant at *p* < 0.05. All experiments were performed in triplicate, and representative results are shown.

## Supplementary Information

**Additional file 1: Figure S1.** DIXDC1-KO mice displayed delayed growth but does not affect fertility. (A) Primer sequence for genotyping DIXDC1 knockout mice (B6.129-Dixdc1tm1Bnrc/J). (B) Result of standard PCR genotyping. WT has band size of 236 bp, homozygote at ~300bp and heterozygote at both 236bp and ~300bp. (C) Mendelian ratio of mice according to the genotype of DIXDC1. *n=196 (*D) Number of pups per female according to their genotype. Number of pups between WT and homozygote is comparable regardless of the genotype of males. *n=1*0 per phenotype. (E) Body weight of WT and DIXDC1 knockout mice from postnatal day 5.5 to 30.5. Body weight of DIXDC1 knockout mice is lighter than its WT counterparts. *n=2*0 per phenotype. (F) Picture of WT and DIXDC1 knockout mice at postnatal day 5.5 and 10.5. Body size of DIXDC1 knockout mice is relatively smaller than WT mice. *p<0.05, **p<0.005 and p***<0.0001, by paired, 2-tailed Student’s *t* test and one-way ANOVA. Error bars represent the mean ± SD. Individual values can be found in Additional file 6: Fig. S1.

**Additional file 2: Figure S2.** DIXDC1 expression in OEC and EPC, and suppression of DIXDC1 expression in HUVEC. (A) Affymetrix gene chip analysis of UCB-MNCs revealed that the expression of DIXDC1 was significantly increased when differentiated into outgrowth ECs from hematopoietic monocytes. (B) DIXDC1 was highly expressed in the OEC stage compared with the UCB-MNC stage, which was confirmed by RT-qPCR. (C) Sequence of DIXDC1 siRNA. (D) qPCR primer sequence. (E) DIXDC1 mRNA expression in HUVEC was silenced by using siRNA with different sequences in concentration dependent manner. (F) DIXDC1 mRNA expression was silenced by using siRNA #2 and #4 in time dependent manner. (G). DIXDC1 siRNA #4 was used to transfect HUVEC in concentration and time dependent manner and protein levels were assessed by using western blot. (H) and (I) Quantification of DIXDC1 level of Fig (G). All Experiments were repeated at least 4 different sets. *p<0.05, **p<0.005 and p***<0.0001, by paired, 2-tailed Student’s *t* test and one-way ANOVA. Error bars represent the mean ± SD. Individual values can be found in Additional file 6: Fig. S2.

**Additional file 3: Figure S3.** Retinae of DIXDC1-KO mice has lower expression of Vegfr2 in filopodia. (A) Mice retinae at postnatal day 9.5 were isolated and immunostained with antibodies against CD31 and VEGFR2. Filopodia of Dixdc1 retinae showed significant decrease in Vegfr2 expression. (B) Quantification of Fig (A). Scale bars: 50μm All Experiments were repeated at least 3 different sets of WT and DIXDC1-KO littermates. *P<0.05, **P<0.005, and ***P<0.0001, by paired, 2-tailed Student’s *t* test. Error bars represent the mean ± SD. Individual values can be found in Additional file 6: Fig. S3.

**Additional file 4: Figure S4.** DIXDC1 upregulate Dvl2 level and further increase basal VEGFR2 level. (A) Dvl2 or Control vector, and VEGFR2 was co-transfected in HEK293T. Immunoprecipitation with antibody against Dvl2 and VEGFR2 result revealed that there is an interaction between Dvl2 and VEGFR2. (B) DIXDC1, Dvl2 and VEGFR2 vectors are transfected in HEK293T and downstream signaling was observed. All Experiments were repeated at least 5 different sets. (C) (D) and (E) Quantification of VEGFR2, p-ERK and p-AKT of Fig (B). All Experiments were repeated at least 4 different sets. *p<0.05, **p<0.005 and p***<0.0001, by one-way ANOVA. Error bars represent the mean ± SD. Individual values can be found in Additional file 6: Fig. S4.

**Additional file 5: Figure S5.** DIXDC1 upregulate VEGFR2 to induce VEGFR2 downstream signaling. (A) conbination of Wnt3a and VEGFR2 upregulate DIXDC1 level and further increase VEGFR2 level in EC. (B)(C)(D) and (E) Quantification of relative intensity of p-VEGFR2(Y1175), VEGFR2, p-ERK1/2 and DIXDC1 of Fig (A). (F) VEGFR2 level is depended on the level of DIXDC1 and Dvl2. (G) Quantification of relative intensity of VEGFR2 of Fig (F). All Experiments were repeated at least 4 different sets. *p<0.05, **p<0.005 and p***<0.0001, by one-way ANOVA. Error bars represent the mean ± SD. Individual values can be found in Additional file 6: Fig. S5.

**Additional file 6.** Individual Value

**Additional file 7.** Uncropped Image

## Data Availability

All data needed to evaluate the conclusions in this paper are present in this paper and the Supplementary Materials. All data generated or analyzed during this study are included in this published article and its supplementary information files. Additional data related to this paper may be requested from Y-GK (ygkwon@yonsei.ac.kr).

## Abbreviations

DIXDC1: DIX domain containing 1
Dvl2: Dishevelled segment polarity protein2
VEGF: Vascular endothelial growth factor
VEGFR2: Vascular endothelial growth factor receptor2
OGD: Oxygen glucose deprivation
OIR: Oxygen-induced retinopathy
HUVEC: Human umbilical vein endothelial cell
EC: Endothelial cell
OEC: Outgrowth endothelial cell
UCB-MNC: Human umbilical cord blood-derived mononuclear cell

## References

[1] Falcon BL, Chintharlapalli S, Uhlik MT, Pytowski B (2016). Antagonist antibodies to vascular endothelial growth factor receptor 2 (VEGFR-2) as anti-angiogenic agents. Pharmacol Therapeut..

[2] Chatterjee S, Heukamp LC, Siobal M, Schottle J, Wieczorek C, Peifer M (2013). Tumor VEGF:VEGFR2 autocrine feed-forward loop triggers angiogenesis in lung cancer. J Clin Invest..

[3] Shibuya M (2014). VEGF-VEGFR signals in health and disease. Biomol Ther (Seoul)..

[4] Abhinand CS, Raju R, Soumya SJ, Arya PS, Sudhakaran PR (2016). VEGF-A/VEGFR2 signaling network in endothelial cells relevant to angiogenesis. J Cell Commun Signal..

[5] Shibuya M (2011). Vascular endothelial growth factor (VEGF) and its receptor (VEGFR) signaling in angiogenesis: a crucial target for anti- and pro-angiogenic therapies. Genes Cancer..

[6] Welti J, Loges S, Dimmeler S, Carmeliet P (2013). Recent molecular discoveries in angiogenesis and antiangiogenic therapies in cancer. J Clin Invest..

[7] Karaman S, Leppanen VM, Alitalo K. Vascular endothelial growth factor signaling in development and disease. Development. 2018;145(14):dev151019. 10.1242/dev.151019 30030240

[8] Marech I, Leporini C, Ammendola M, Porcelli M, Gadaleta CD, Russo E (2016). Classical and non-classical proangiogenic factors as a target of antiangiogenic therapy in tumor microenvironment. Cancer Lett..

[9] Eilken HM, Adams RH (2010). Dynamics of endothelial cell behavior in sprouting angiogenesis. Curr Opin Cell Biol..

[10] Gerhardt H, Golding M, Fruttiger M, Ruhrberg C, Lundkvist A, Abramsson A (2003). VEGF guides angiogenic sprouting utilizing endothelial tip cell filopodia. J Cell Biol..

[11] Hellstrom M, Phng LK, Hofmann JJ, Wallgard E, Coultas L, Lindblom P (2007). Dll4 signalling through Notch1 regulates formation of tip cells during angiogenesis. Nature..

[12] Hellstrom M, Phng LK, Gerhardt H (2007). VEGF and Notch signaling: the yin and yang of angiogenic sprouting. Cell Adh Migr..

[13] Blanco R, Gerhardt H (2013). VEGF and Notch in tip and stalk cell selection. Cold Spring Harb Perspect Med..

[14] Dejana E (2010). The role of wnt signaling in physiological and pathological angiogenesis. Circ Res..

[15] Du J, Li J (2018). The role of Wnt signaling pathway in atherosclerosis and its relationship with angiogenesis. Exp Ther Med..

[16] Parmalee NL, Kitajewski J (2008). Wnt signaling in angiogenesis. Curr Drug Targets..

[17] Olsen JJ, Pohl S, Deshmukh A, Visweswaran M, Ward NC, Arfuso F (2017). The role of Wnt signalling in angiogenesis. Clin Biochem Rev..

[18] Martowicz A, Trusohamn M, Jensen N, Wisniewska-Kruk J, Corada M, Ning FC (2019). Endothelial beta-catenin signaling supports postnatal brain and retinal angiogenesis by promoting sprouting, tip cell formation, and VEGFR (Vascular Endothelial Growth Factor Receptor) 2 expression. Arterioscler Thromb Vasc Biol..

[19] Liu YT, Dan QJ, Wang J, Feng Y, Chen L, Liang J (2011). Molecular basis of Wnt activation via the DIX domain protein Ccd1. J Biol Chem..

[20] Terawaki SI, Fujita S, Katsutani T, Shiomi K, Keino-Masu K, Masu M (2017). Structural basis for Ccd1 auto-inhibition in the Wnt pathway through homomerization of the DIX domain. Sci Rep..

[21] Li X, Xiao Y, Fan S, Xiao M, Wang X, Zhu X (2016). Overexpression of DIXDC1 correlates with enhanced cell growth and poor prognosis in human pancreatic ductal adenocarcinoma. Hum Pathol..

[22] Xu Z, Liu D, Fan C, Luan L, Zhang X, Wang E (2014). DIXDC1 increases the invasion and migration ability of non-small-cell lung cancer cells via the PI3K-AKT/AP-1 pathway. Mol Carcinog..

[23] Wang L, Cao XX, Chen Q, Zhu TF, Zhu HG, Zheng L (2009). DIXDC1 targets p21 and cyclin D1 via PI3K pathway activation to promote colon cancer cell proliferation. Cancer Sci..

[24] Tan C, Qiao F, Wei P, Chi Y, Wang W, Ni S (2016). DIXDC1 activates the Wnt signaling pathway and promotes gastric cancer cell invasion and metastasis. Mol Carcinog..

[25] Zhou S, Shen J, Lin S, Liu X, Xu M, Shi L (2016). Downregulated expression of DIXDC1 in hepatocellular carcinoma and its correlation with prognosis. Tumour Biol..

[26] Kwan V, Meka DP, White SH, Hung CL, Holzapfel NT, Walker S (2016). DIXDC1 phosphorylation and control of dendritic morphology are impaired by rare genetic variants. Cell Rep..

[27] Kivimae S, Martin PM, Kapfhamer D, Ruan Y, Heberlein U, Rubenstein JL (2011). Abnormal behavior in mice mutant for the Disc1 binding partner, Dixdc1. Transl Psychiatry..

[28] Wu W, Liu Q, Liu Y, Yu Z, Wang Y (2016). Dixdc1 targets CyclinD1 and p21 via PI3K pathway activation to promote Schwann cell proliferation after sciatic nerve crush. Biochem Biophys Res Commun..

[29] Zhou XH, Lin W, Ren YM, Liu S, Fan BY, Wei ZJ (2017). Comparison of DNA methylation in Schwann cells before and after peripheral nerve injury in rats. Biomed Res Int..

[30] Maeng YS, Choi HJ, Kwon JY, Park YW, Choi KS, Min JK (2009). Endothelial progenitor cell homing: prominent role of the IGF2-IGF2R-PLCbeta2 axis. Blood..

[31] Sobczak M, Dargatz J, Chrzanowska-Wodnicka M. Isolation and culture of pulmonary endothelial cells from neonatal mice. J Vis Exp. 2010;46:2316. 10.3791/2316 PMC3278331 21178973

[32] Smalley MJ, Signoret N, Robertson D, Tilley A, Hann A, Ewan K (2005). Dishevelled (Dvl-2) activates canonical Wnt signalling in the absence of cytoplasmic puncta. J Cell Sci..

[33] Prahst C, Heroult M, Lanahan AA, Uziel N, Kessler O, Shraga-Heled N (2008). Neuropilin-1-VEGFR-2 complexing requires the PDZ-binding domain of neuropilin-1. J Biol Chem..

[34] Tae N, Lee S, Kim O, Park J, Na S, Lee JH (2017). Syntenin promotes VEGF-induced VEGFR2 endocytosis and angiogenesis by increasing ephrin-B2 function in endothelial cells. Oncotarget..

[35] Xu W, Zhou W, Cheng M, Wang J, Liu Z, He S (2017). Hypoxia activates Wnt/beta-catenin signaling by regulating the expression of BCL9 in human hepatocellular carcinoma. Sci Rep..

[36] Tang N, Wang L, Esko J, Giordano FJ, Huang Y, Gerber HP (2004). Loss of HIF-1alpha in endothelial cells disrupts a hypoxia-driven VEGF autocrine loop necessary for tumorigenesis. Cancer Cell..

[37] Benedito R, Rocha SF, Woeste M, Zamykal M, Radtke F, Casanovas O (2012). Notch-dependent VEGFR3 upregulation allows angiogenesis without VEGF-VEGFR2 signalling. Nature..

[38] Gammons MV, Renko M, Johnson CM, Rutherford TJ, Bienz M (2016). Wnt signalosome assembly by DEP domain swapping of dishevelled. Mol Cell..

[39] Yoshihara T, Takahashi-Yanaga F, Shiraishi F, Morimoto S, Watanabe Y, Hirata M (2010). Anti-angiogenic effects of differentiation-inducing factor-1 involving VEGFR-2 expression inhibition independent of the Wnt/beta-catenin signaling pathway. Mol Cancer..

[40] Phng LK, Potente M, Leslie JD, Babbage J, Nyqvist D, Lobov I (2009). Nrarp coordinates endothelial Notch and Wnt signaling to control vessel density in angiogenesis. Dev Cell..

[41] Easwaran V, Lee SH, Inge L, Guo L, Goldbeck C, Garrett E (2003). beta-Catenin regulates vascular endothelial growth factor expression in colon cancer. Cancer Res..

[42] Gao C, Chen YG (2010). Dishevelled: The hub of Wnt signaling. Cell Signal..

[43] Gonzalez-Sancho JM, Greer YE, Abrahams CL, Takigawa Y, Baljinnyam B, Lee KH (2013). Functional consequences of Wnt-induced dishevelled 2 phosphorylation in canonical and noncanonical Wnt signaling. J Biol Chem..

[44] Smith GA, Fearnley GW, Abdul-Zani I, Wheatcroft SB, Tomlinson DC, Harrison MA (2017). Ubiquitination of basal VEGFR2 regulates signal transduction and endothelial function. Biol Open..

[45] Barben M, Schori C, Samardzija M, Grimm C (2018). Targeting Hif1a rescues cone degeneration and prevents subretinal neovascularization in a model of chronic hypoxia. Mol Neurodegener..

[46] Lin M, Chen Y, Jin J, Hu Y, Zhou KK, Zhu M (2011). Ischaemia-induced retinal neovascularisation and diabetic retinopathy in mice with conditional knockout of hypoxia-inducible factor-1 in retinal Muller cells. Diabetologia..

[47] Gao Y, Jing M, Ge R, Lang L (2016). Induction of hypoxia-inducible factor-1alpha by BDNF protects retinoblastoma cells against chemotherapy-induced apoptosis. Mol Cell Biochem..

[48] Liu N, Sun Y, Zhao N, Chen L (2014). Role of hypoxia-inducible factor-1alpha and survivin in oxygen-induced retinopathy in mice. Int J Clin Exp Pathol..

[49] Sun Z, Li X, Massena S, Kutschera S, Padhan N, Gualandi L (2012). VEGFR2 induces c-Src signaling and vascular permeability in vivo via the adaptor protein TSAd. J Exp Med..

[50] Li T, Wan YC, Sun LJ, Tao SJ, Chen P, Liu CH (2018). DIXDC1 prevents oxygen-glucose deprivation/reoxygenation-induced injury in hippocampal neurons in vitro by promoting Wnt/beta-catenin signaling. Eur Rev Med Pharmacol Sci..

[51] Lu H, Jiang R, Tao X, Duan C, Huang J, Huan W (2017). Expression of Dixdc1 and its role in astrocyte proliferation after traumatic brain injury. Cell Mol Neurobiol..

[52] Zhang Q, Bai X, Chen W, Ma T, Hu Q, Liang C (2013). Wnt/beta-catenin signaling enhances hypoxia-induced epithelial-mesenchymal transition in hepatocellular carcinoma via crosstalk with hif-1alpha signaling. Carcinogenesis..

[53] Liu Z, Qi L, Li Y, Zhao X, Sun B (2017). VEGFR2 regulates endothelial differentiation of colon cancer cells. BMC Cancer..

[54] Marin V, Kaplanski G, Gres S, Farnarier C, Bongrand P (2001). Endothelial cell culture: protocol to obtain and cultivate human umbilical endothelial cells. J Immunol Methods..

[55] Choi YS, Choi HJ, Min JK, Pyun BJ, Maeng YS, Park H (2009). Interleukin-33 induces angiogenesis and vascular permeability through ST2/TRAF6-mediated endothelial nitric oxide production. Blood..

[56] DeCicco-Skinner KL, Henry GH, Cataisson C, Tabib T, Gwilliam JC, Watson NJ (2014). Endothelial cell tube formation assay for the in vitro study of angiogenesis. J Vis Exp..

[57] Nakatsu MN, Davis J, Hughes CC (2007). Optimized fibrin gel bead assay for the study of angiogenesis. J Vis Exp..

[58] Baker M, Robinson SD, Lechertier T, Barber PR, Tavora B, D'Amico G (2012). Use of the mouse aortic ring assay to study angiogenesis. Nat Protoc..

[59] Connor KM, Krah NM, Dennison RJ, Aderman CM, Chen J, Guerin KI (2009). Quantification of oxygen-induced retinopathy in the mouse: a model of vessel loss, vessel regrowth and pathological angiogenesis. Nat Protoc..

[60] Dunn L, Prosser HC, Tan JT, Vanags LZ, Ng MK, Bursill CA (2013). Murine model of wound healing. J Vis Exp..

