# Supplementary material for: DIX domain containing 1 (DIXDC1) modulates VEGFR2 level in vasculatures to regulate embryonic and postnatal retina angiogenesis
Source: BMC Biol. 2022 Feb 10;20:41. doi: 10.1186/s12915-022-01240-3 (PMC8830128; doi:10.1186/s12915-022-01240-3)

A

Primer Type	Sequence(5'→3')
Mutant Forward	CTC GTG CTT TAC GGT ATC GC
Common	ACT GTT GTC TGG GTG ATG GA
WT Reverse	ACC TTT GGG AGG AAC TGT TGA

B

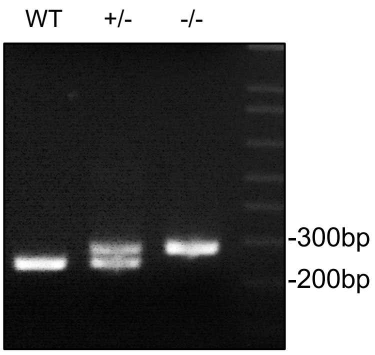

C

DIXDC1 Genotype (196 animals)	Expected Number	Observed Number
Wild Type	49 (25%)	46 (23.5%)
Heterozygote	98 (50%)	102 (52.0%)
Homozygote	49 (25%)	48 (24.5%)

D

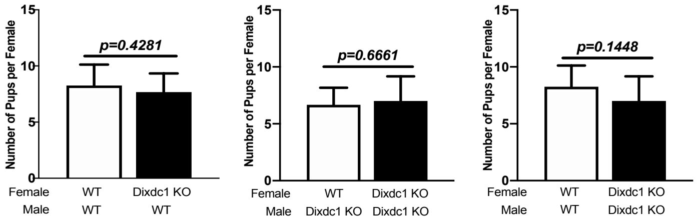

E

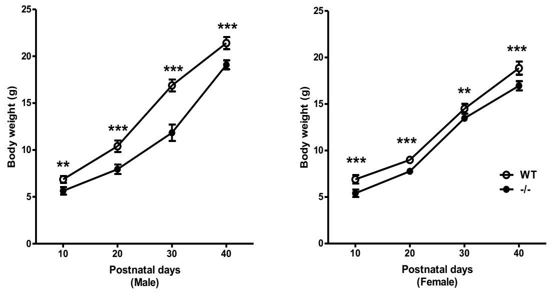

F

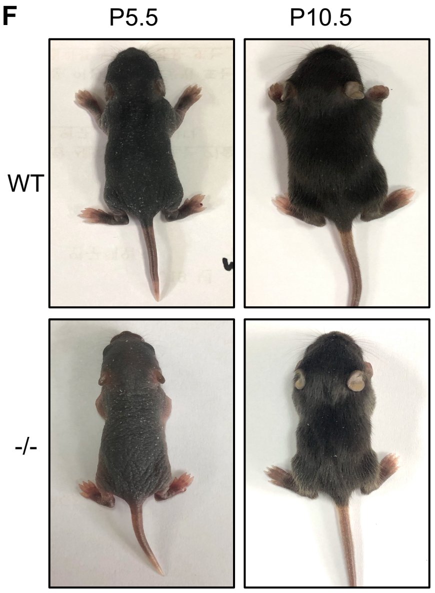

Supplement: Supplementary file 1 — Additional file 1: Figure S1. DIXDC1-KO mice displayed delayed growth but does not affect fertility. (A) Primer sequence for genotyping DIXDC1 knockout mice (B6.129-Dixdc1tm1Bnrc/J). (B) Result of standard PCR genotyping. WT has band size of 236 bp, homozygote at ~300bp and heterozygote at both 236bp and ~300bp. (C) Mendelian ratio of mice according to the genotype of DIXDC1. n=196 (D) Number of pups per female according to their genotype. Number of pups between WT and homozygote is comparable regardless of the genotype of males. n=10 per phenotype. (E) Body weight of WT and DIXDC1 knockout mice from postnatal day 5.5 to 30.5. Body weight of DIXDC1 knockout mice is lighter than its WT counterparts. n=20 per phenotype. (F) Picture of WT and DIXDC1 knockout mice at postnatal day 5.5 and 10.5. Body size of DIXDC1 knockout mice is relatively smaller than WT mice. *p<0.05, **p<0.005 and p***<0.0001, by paired, 2-tailed Student’s t test and one-way ANOVA. Error bars represent the mean ± SD. Individual values can be found in Additional file 6: Fig. S1. [file 12915_2022_1240_MOESM1_ESM.pdf]
