# Supplementary material for: DIX domain containing 1 (DIXDC1) modulates VEGFR2 level in vasculatures to regulate embryonic and postnatal retina angiogenesis
Source: BMC Biol. 2022 Feb 10;20:41. doi: 10.1186/s12915-022-01240-3 (PMC8830128; doi:10.1186/s12915-022-01240-3)

A

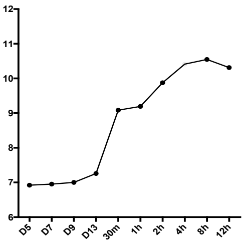

B

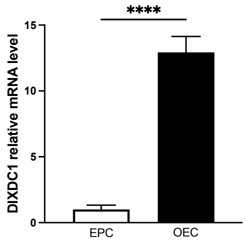

C

siRNA no.	Target Sequence
#1(D-007758-01)	GUACUAAAGUGCUCUAUUU
#2(D-007758-02)	GCAAAGAGCGAGUCCAUUA
#3(D-007758-03)	GGGCAAAUAUGGACAAAGA
#4(D-007758-04)	GGAAGGAAAUCACCGGUAU

D

Gene	Sense (5'→3')	Antisense (5'→3')
Human VEGFR2	CTCAAGACAGGAAGACCAAG	ACTGCTACTGTCCTG CAAGT
Human DIXDC1	GGGCTTCAAATGAGCAACAGC	CCATCCCGGAGATCTTGTCG
Human GAPDH	CATGTACGTTGCTATCCAGGC	CTCCTTAATGTCACGCACGAT

E

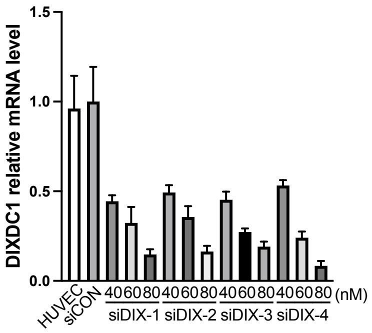

F

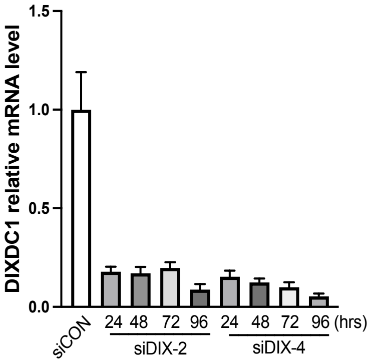

G

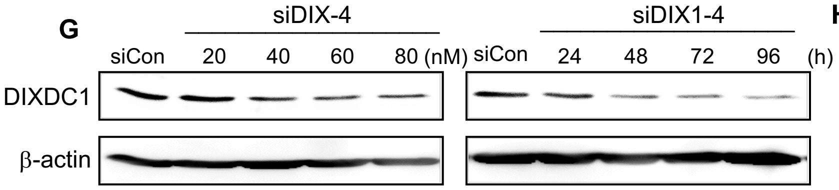

H

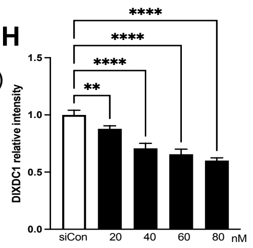

I

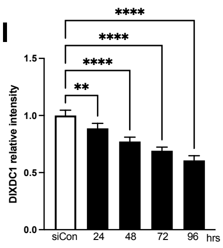

Supplement: Supplementary file 2 — Additional file 2: Figure S2. DIXDC1 expression in OEC and EPC, and suppression of DIXDC1 expression in HUVEC. (A) Affymetrix gene chip analysis of UCB-MNCs revealed that the expression of DIXDC1 was significantly increased when differentiated into outgrowth ECs from hematopoietic monocytes. (B) DIXDC1 was highly expressed in the OEC stage compared with the UCB-MNC stage, which was confirmed by RT-qPCR. (C) Sequence of DIXDC1 siRNA. (D) qPCR primer sequence. (E) DIXDC1 mRNA expression in HUVEC was silenced by using siRNA with different sequences in concentration dependent manner. (F) DIXDC1 mRNA expression was silenced by using siRNA #2 and #4 in time dependent manner. (G). DIXDC1 siRNA #4 was used to transfect HUVEC in concentration and time dependent manner and protein levels were assessed by using western blot. (H) and (I) Quantification of DIXDC1 level of Fig (G). All Experiments were repeated at least 4 different sets. *p<0.05, **p<0.005 and p***<0.0001, by paired, 2-tailed Student’s t test and one-way ANOVA. Error bars represent the mean ± SD. Individual values can be found in Additional file 6: Fig. S2. [file 12915_2022_1240_MOESM2_ESM.pdf]
