# Supplementary figures and images for: DIX domain containing 1 (DIXDC1) modulates VEGFR2 level in vasculatures to regulate embryonic and postnatal retina angiogenesis
Source: BMC Biol. 2022 Feb 10;20:41. doi: 10.1186/s12915-022-01240-3 (PMC8830128; doi:10.1186/s12915-022-01240-3)

**A**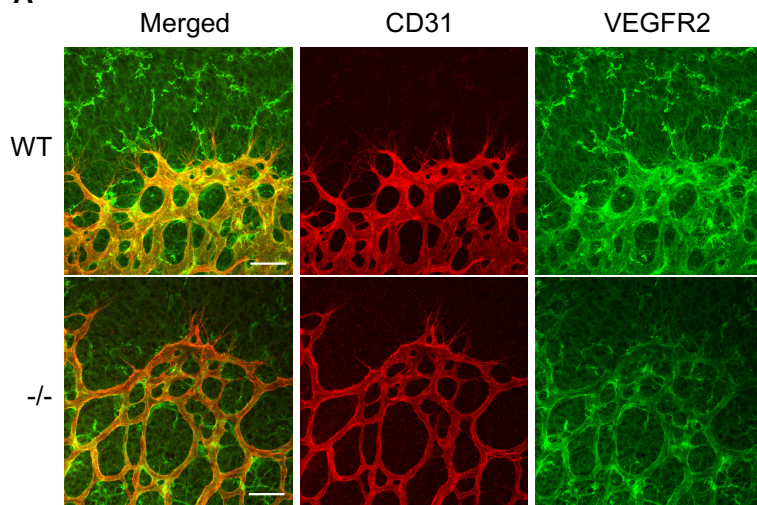**B**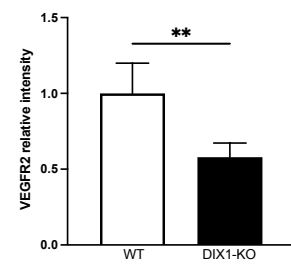

Supplement: Supplementary file 3 — Additional file 3: Figure S3. Retinae of DIXDC1-KO mice has lower expression of Vegfr2 in filopodia. (A) Mice retinae at postnatal day 9.5 were isolated and immunostained with antibodies against CD31 and VEGFR2. Filopodia of Dixdc1 retinae showed significant decrease in Vegfr2 expression. (B) Quantification of Fig (A). Scale bars: 50μm All Experiments were repeated at least 3 different sets of WT and DIXDC1-KO littermates. *P<0.05, **P<0.005, and ***P<0.0001, by paired, 2-tailed Student’s t test. Error bars represent the mean ± SD. Individual values can be found in Additional file 6: Fig. S3. [file 12915_2022_1240_MOESM3_ESM.pdf]

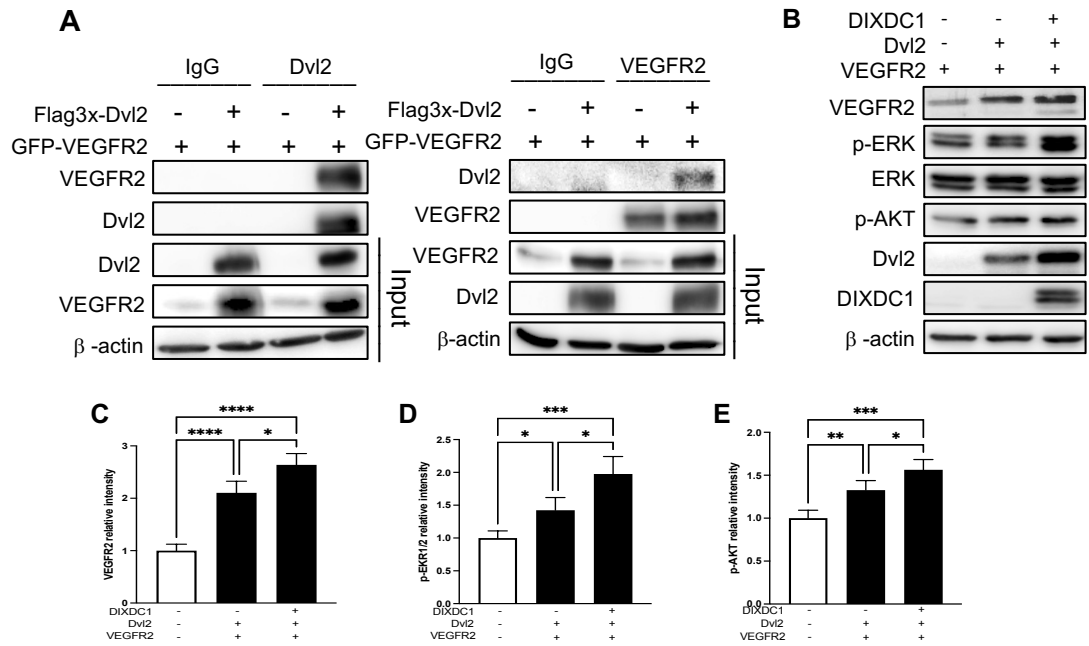

Supplement: Supplementary file 4 — Additional file 4: Figure S4. DIXDC1 upregulate Dvl2 level and further increase basal VEGFR2 level. (A) Dvl2 or Control vector, and VEGFR2 was co-transfected in HEK293T. Immunoprecipitation with antibody against Dvl2 and VEGFR2 result revealed that there is an interaction between Dvl2 and VEGFR2. (B) DIXDC1, Dvl2 and VEGFR2 vectors are transfected in HEK293T and downstream signaling was observed. All Experiments were repeated at least 5 different sets. (C) (D) and (E) Quantification of VEGFR2, p-ERK and p-AKT of Fig (B). All Experiments were repeated at least 4 different sets. *p<0.05, **p<0.005 and p***<0.0001, by one-way ANOVA. Error bars represent the mean ± SD. Individual values can be found in Additional file 6: Fig. S4. [file 12915_2022_1240_MOESM4_ESM.pdf]

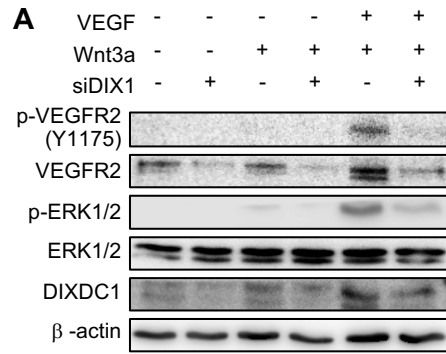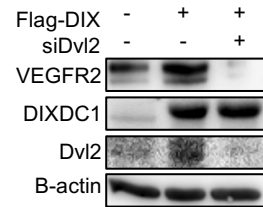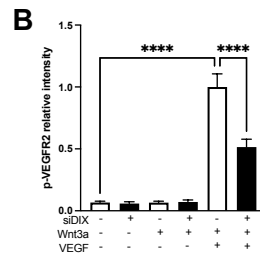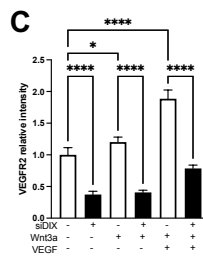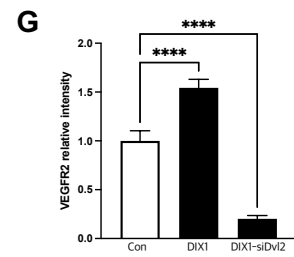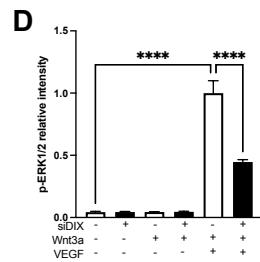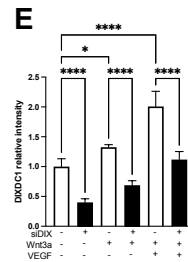

Supplement: Supplementary file 5 — Additional file 5: Figure S5. DIXDC1 upregulate VEGFR2 to induce VEGFR2 downstream signaling. (A) conbination of Wnt3a and VEGFR2 upregulate DIXDC1 level and further increase VEGFR2 level in EC. (B)(C)(D) and (E) Quantification of relative intensity of p-VEGFR2(Y1175), VEGFR2, p-ERK1/2 and DIXDC1 of Fig (A). (F) VEGFR2 level is depended on the level of DIXDC1 and Dvl2. (G) Quantification of relative intensity of VEGFR2 of Fig (F). All Experiments were repeated at least 4 different sets. *p<0.05, **p<0.005 and p***<0.0001, by one-way ANOVA. Error bars represent the mean ± SD. Individual values can be found in Additional file 6: Fig. S5. [file 12915_2022_1240_MOESM5_ESM.pdf]
