# Supplementary material for: DIX domain containing 1 (DIXDC1) modulates VEGFR2 level in vasculatures to regulate embryonic and postnatal retina angiogenesis
Source: BMC Biol. 2022 Feb 10;20:41. doi: 10.1186/s12915-022-01240-3 (PMC8830128; doi:10.1186/s12915-022-01240-3)

Fig 4

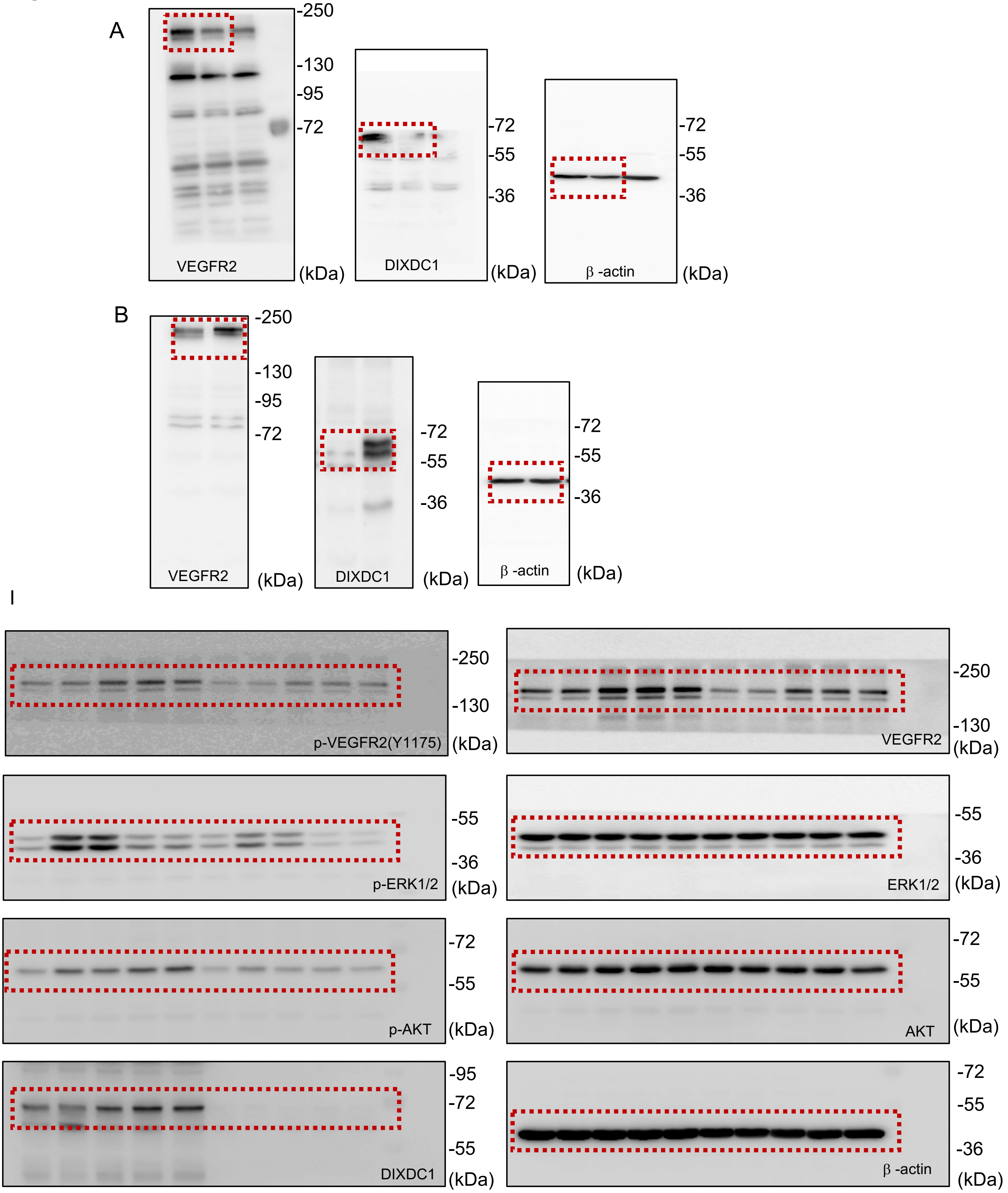
A
-250
-130
-95
-72
VEGFR2
(kDa)
-72
-55
-36
DIXDC1
(kDa)
-72
-55
-36
β -actin
(kDa)
B
-250
-130
-95
-72
VEGFR2
(kDa)
-72
-55
-36
DIXDC1
(kDa)
-72
-55
-36
β -actin
(kDa)
I
-250
-130
p-VEGFR2(Y1175)
(kDa)
-250
-130
VEGFR2
(kDa)
-55
-36
p-ERK1/2
(kDa)
-55
-36
ERK1/2
(kDa)
-72
-55
p-AKT
(kDa)
-72
-55
AKT
(kDa)
-95
-72
-55
DIXDC1
(kDa)
-72
-55
-36
β -actin
(kDa)

Fig 5

A

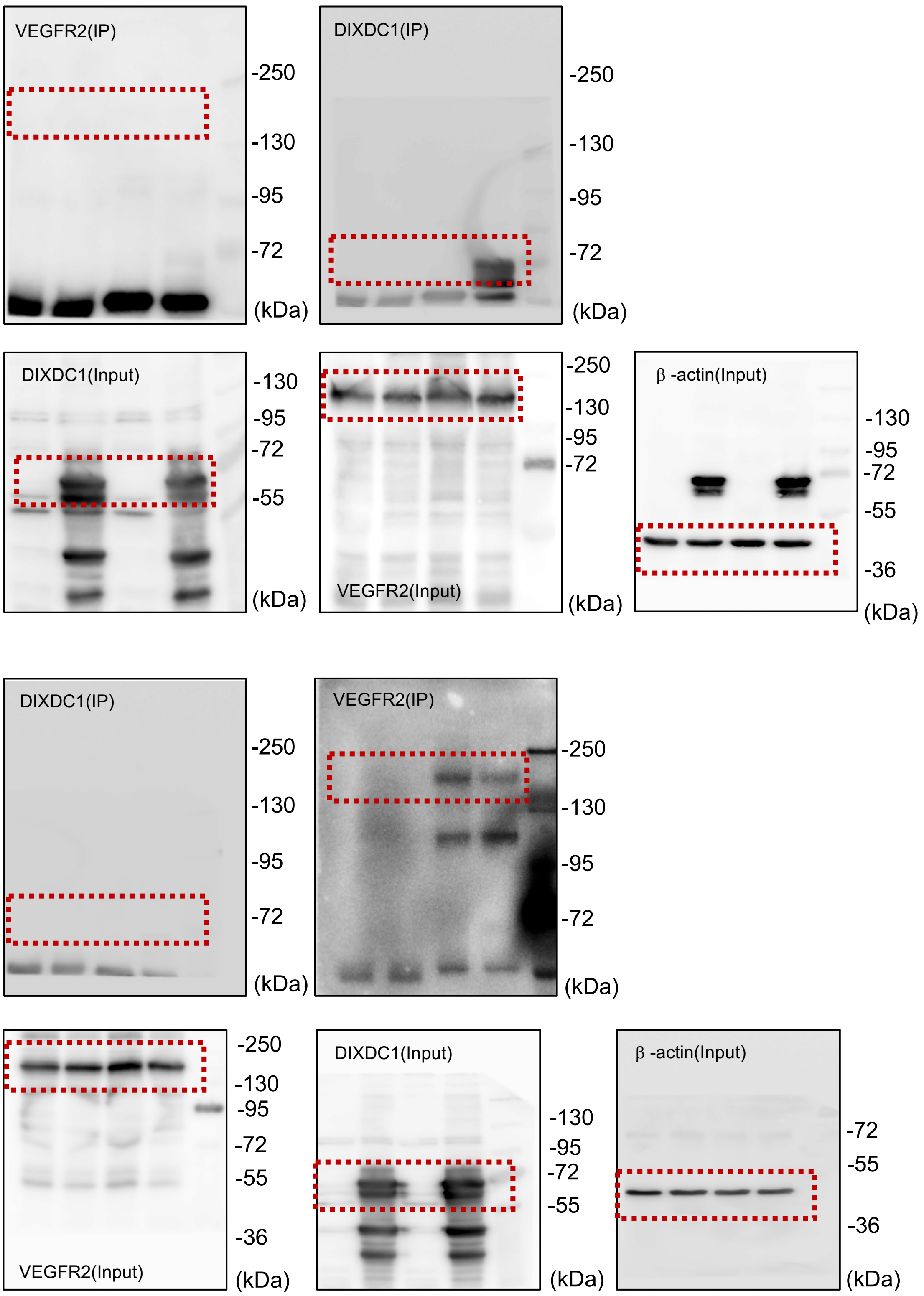
VEGFR2(IP)
-250
-130
-95
-72
(kDa)
DIXDC1(IP)
-250
-130
-95
-72
(kDa)
DIXDC1(Input)
-130
-95
-72
-55
(kDa)
-250
-130
-95
-72
VEGFR2(Input)
(kDa)
β -actin(Input)
-130
-95
-72
-55
-36
(kDa)
DIXDC1(IP)
-250
-130
-95
-72
(kDa)
VEGFR2(IP)
-250
-130
-95
-72
(kDa)
-250
-130
-95
-72
-55
-36
VEGFR2(Input)
(kDa)
DIXDC1(Input)
-130
-95
-72
-55
(kDa)
β -actin(Input)
-72
-55
-36
(kDa)

Fig 5

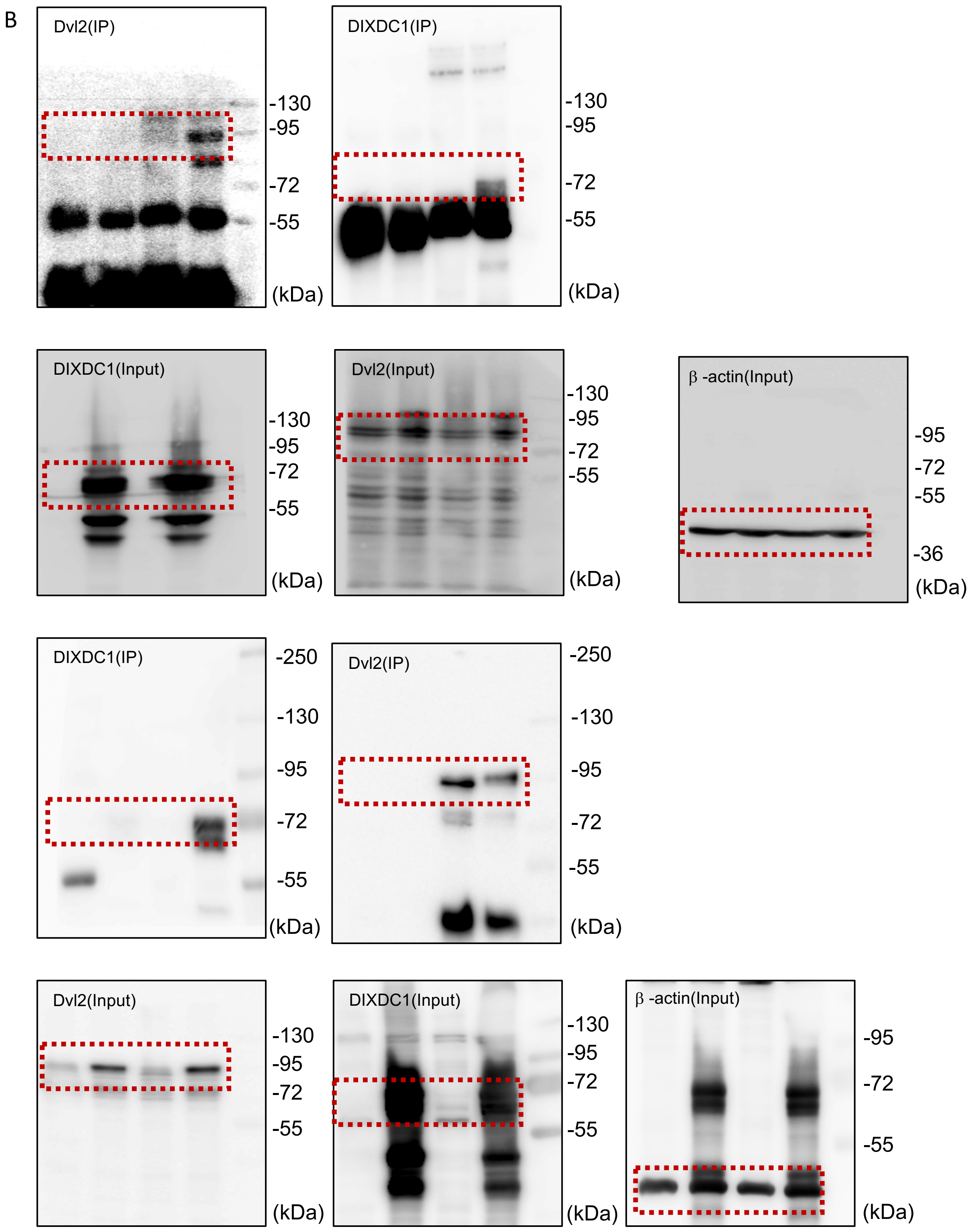

B
Dvl2(IP)
-130
-95
-72
-55
(kDa)
DIXDC1(IP)
-130
-95
-72
-55
(kDa)
DIXDC1(Input)
-130
-95
-72
-55
(kDa)
Dvl2(Input)
-130
-95
-72
-55
(kDa)
β -actin(Input)
-95
-72
-55
-36
(kDa)
DIXDC1(IP)
-250
-130
-95
-72
-55
(kDa)
Dvl2(IP)
-250
-130
-95
-72
-55
(kDa)
Dvl2(Input)
-130
-95
-72
-55
(kDa)
DIXDC1(Input)
-130
-95
-72
-55
(kDa)
β -actin(Input)
-95
-72
-55
(kDa)

Fig 5

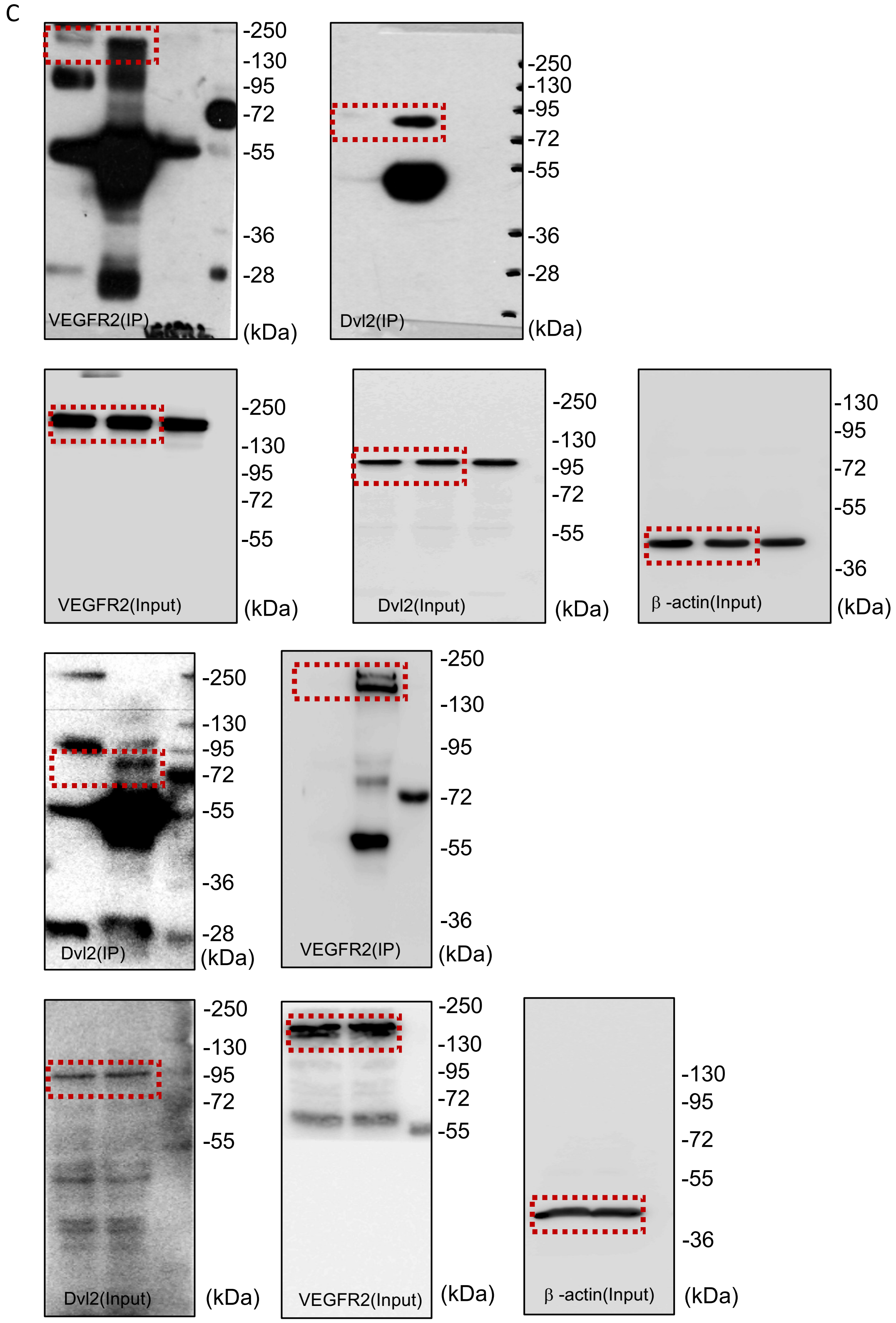
C
-250
-130
-95
-72
-55
-36
-28
VEGFR2(IP)
(kDa)
-250
-130
-95
-72
-55
-36
-28
Dvl2(IP)
(kDa)
-250
-130
-95
-72
-55
VEGFR2(Input)
(kDa)
-250
-130
-95
-72
-55
Dvl2(Input)
(kDa)
-130
-95
-72
-55
-36
β -actin(Input)
(kDa)
-250
-130
-95
-72
-55
-36
-28
Dvl2(IP)
(kDa)
-250
-130
-95
-72
-55
-36
VEGFR2(IP)
(kDa)
-250
-130
-95
-72
-55
Dvl2(Input)
(kDa)
-250
-130
-95
-72
-55
VEGFR2(Input)
(kDa)
-130
-95
-72
-55
-36
β -actin(Input)
(kDa)

Fig 5

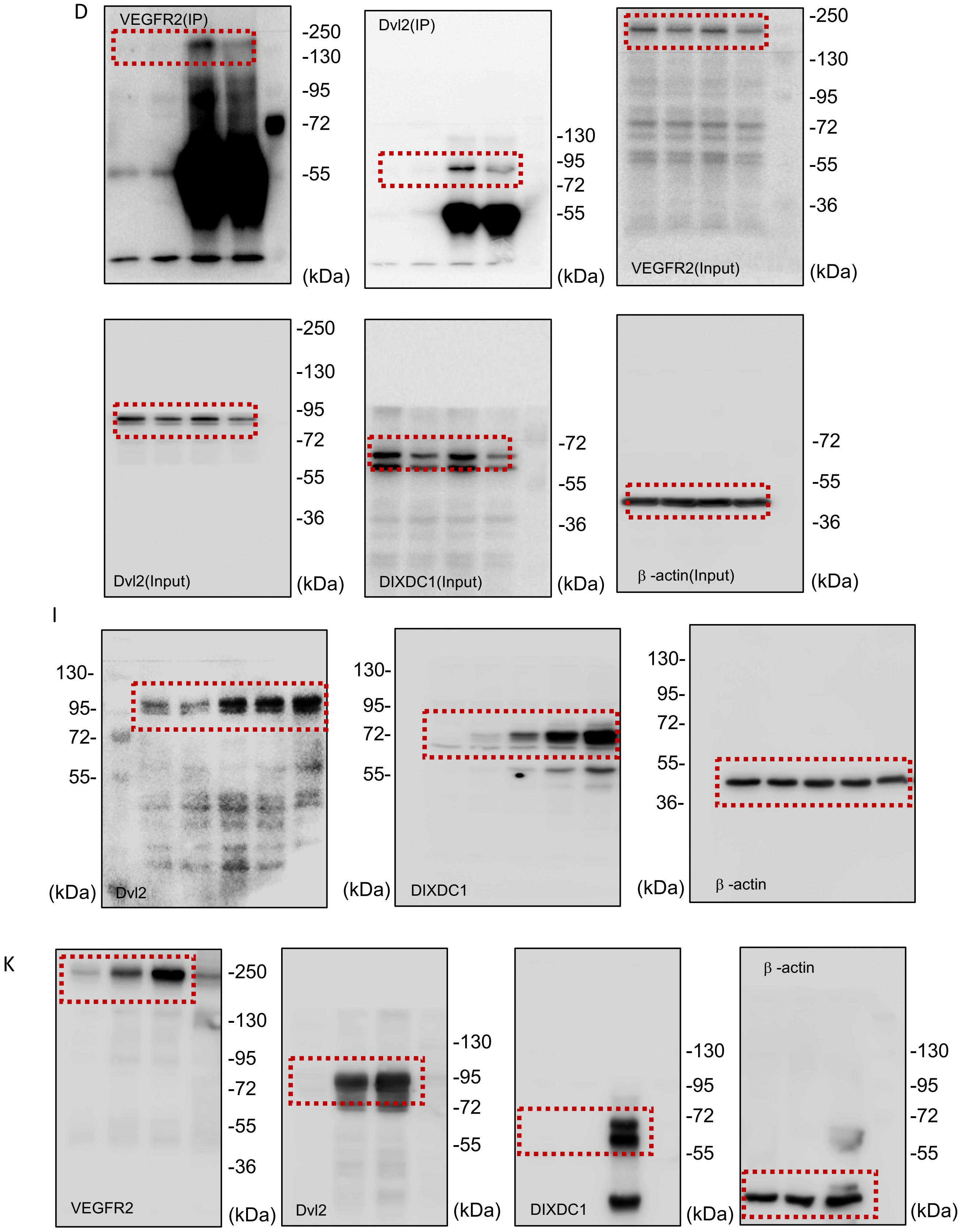
D
VEGFR2(IP)
-250
-130
-95
-72
-55
(kDa)
Dvl2(IP)
-130
-95
-72
-55
(kDa)
-250
-130
-95
-72
-55
-36
VEGFR2(Input)
(kDa)
-250
-130
-95
-72
-55
-36
Dvl2(Input)
(kDa)
-72
-55
-36
DIXDC1(Input)
(kDa)
-72
-55
-36
β -actin(Input)
(kDa)
I
130-
95-
72-
55-
(kDa)
Dvl2
130-
95-
72-
55-
(kDa)
DIXDC1
130-
95-
72-
55-
36-
(kDa)
β -actin
K
-250
-130
-95
-72
-55
-36
VEGFR2
(kDa)
-130
-95
-72
-55
Dvl2
(kDa)
-130
-95
-72
-55
DIXDC1
(kDa)
β -actin
-130
-95
-72
-55
(kDa)

Fig 6

A

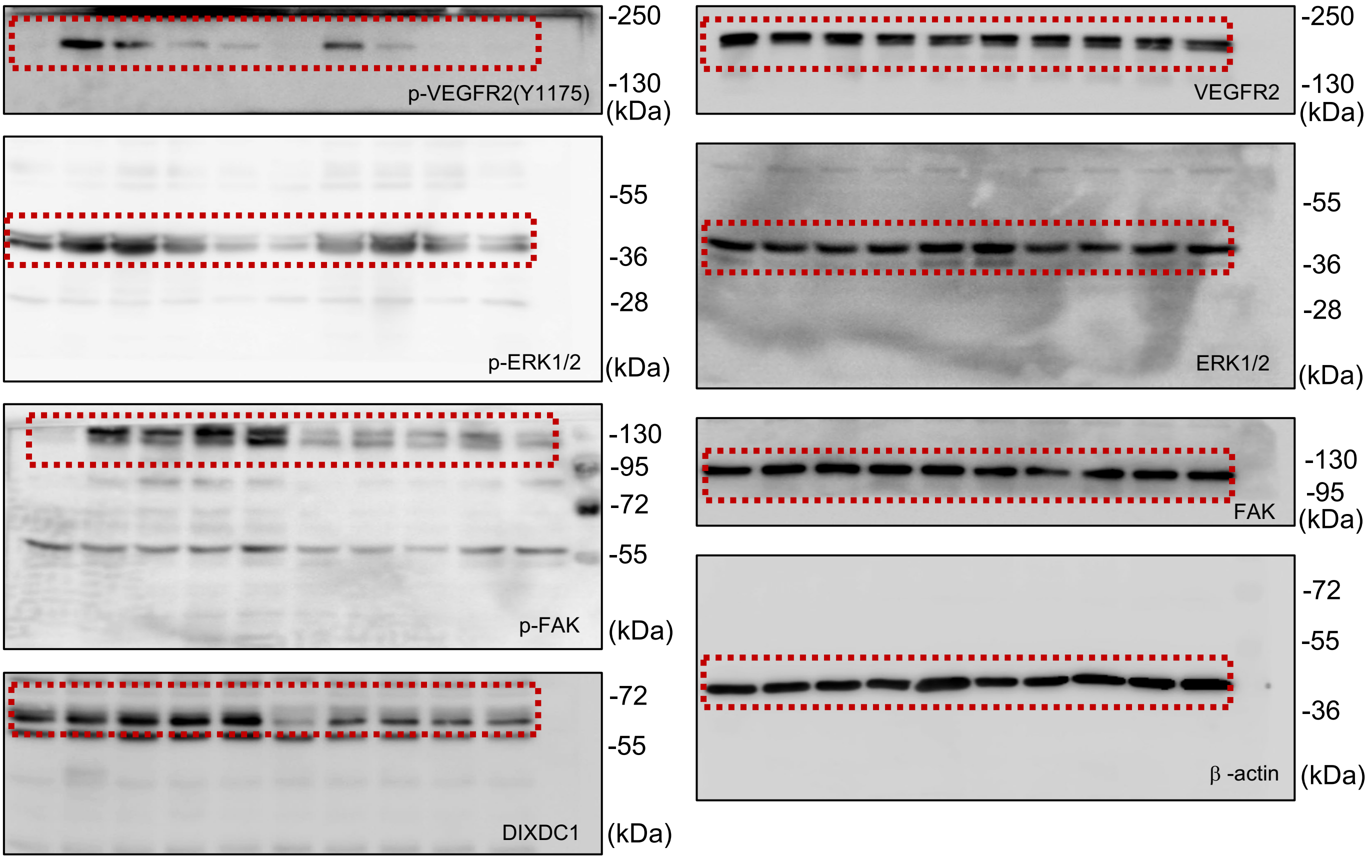
p-VEGFR2(Y1175)
-250
-130
(kDa)
VEGFR2
-250
-130
(kDa)
p-ERK1/2
-55
-36
-28
(kDa)
ERK1/2
-55
-36
-28
(kDa)
p-FAK
-130
-95
-72
-55
(kDa)
FAK
-130
-95
(kDa)
DIXDC1
-72
-55
(kDa)
β -actin
-72
-55
-36
(kDa)

E

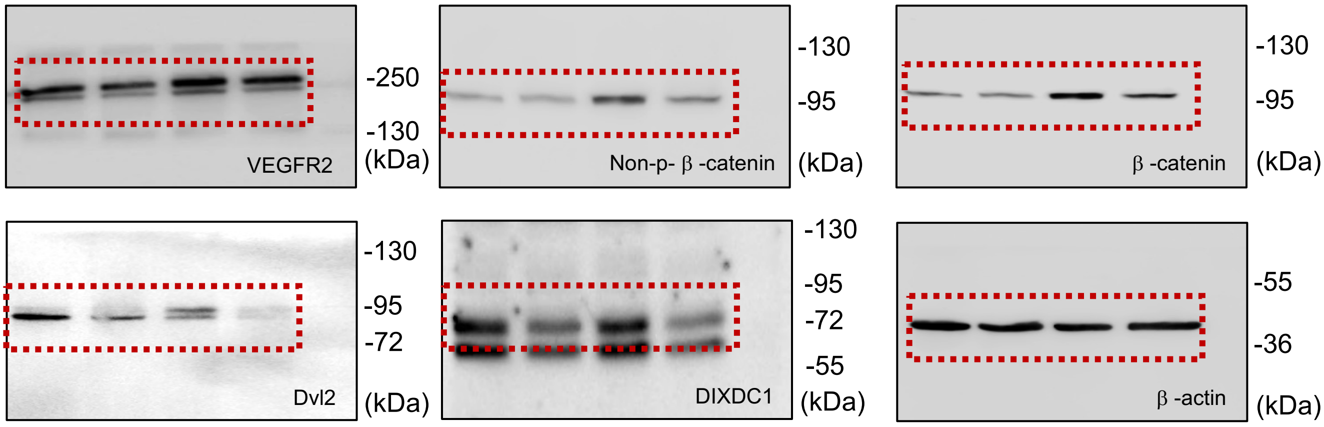
VEGFR2
-250
-130
(kDa)
Non-p- β -catenin
-130
-95
(kDa)
β -catenin
-130
-95
(kDa)
Dvl2
-130
-95
-72
(kDa)
DIXDC1
-130
-95
-72
-55
(kDa)
β -actin
-55
-36
(kDa)

Fig 6

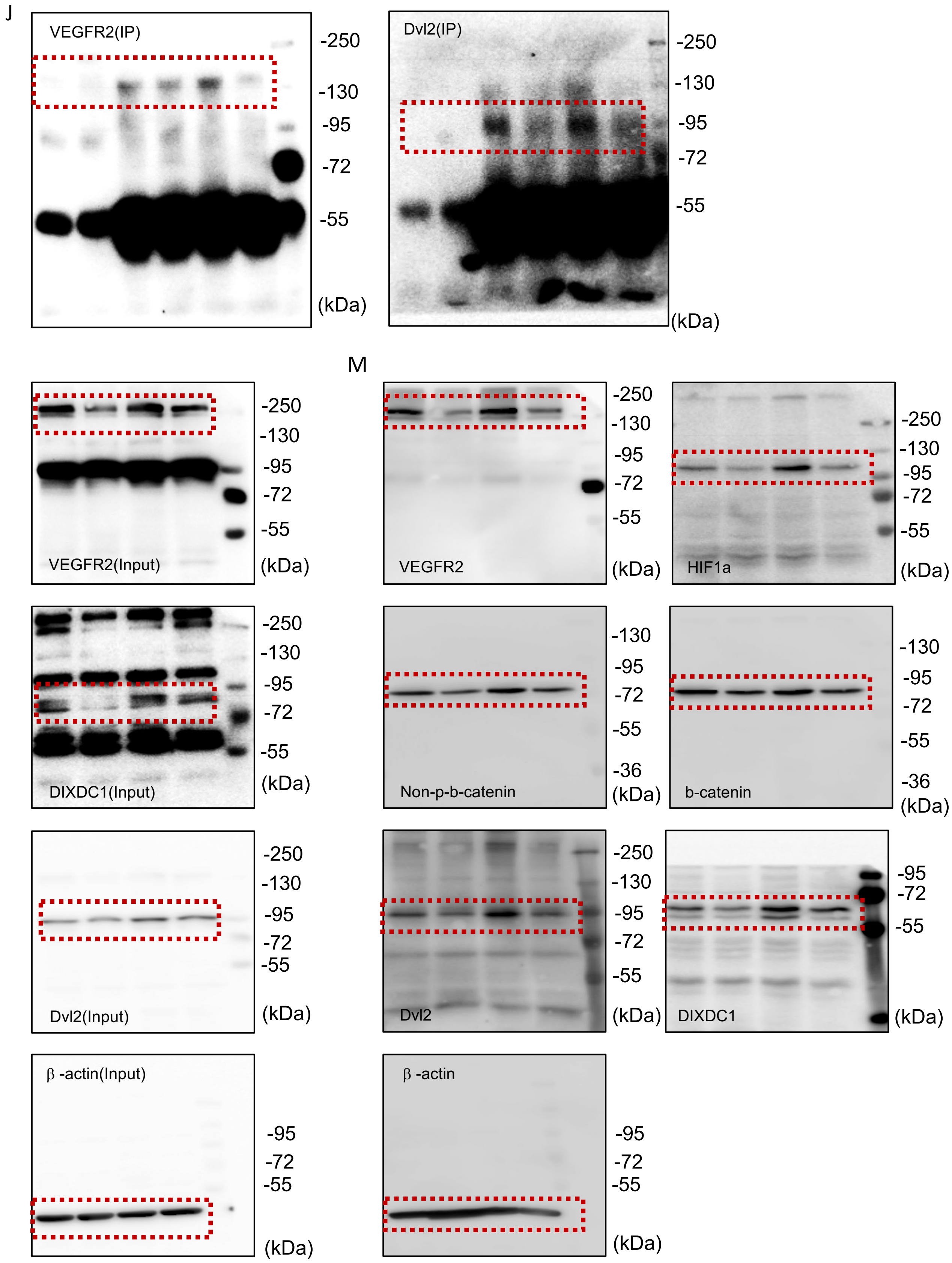
J
VEGFR2(IP)
-250
-130
-95
-72
-55
(kDa)
Dvl2(IP)
-250
-130
-95
-72
-55
(kDa)
VEGFR2(Input)
DIXDC1(Input)
Dvl2(Input)
β -actin(Input)
M
VEGFR2
HIF1a
Non-p-b-catenin
b-catenin
Dvl2
DIXDC1
β -actin
-36
(kDa)

Fig 6

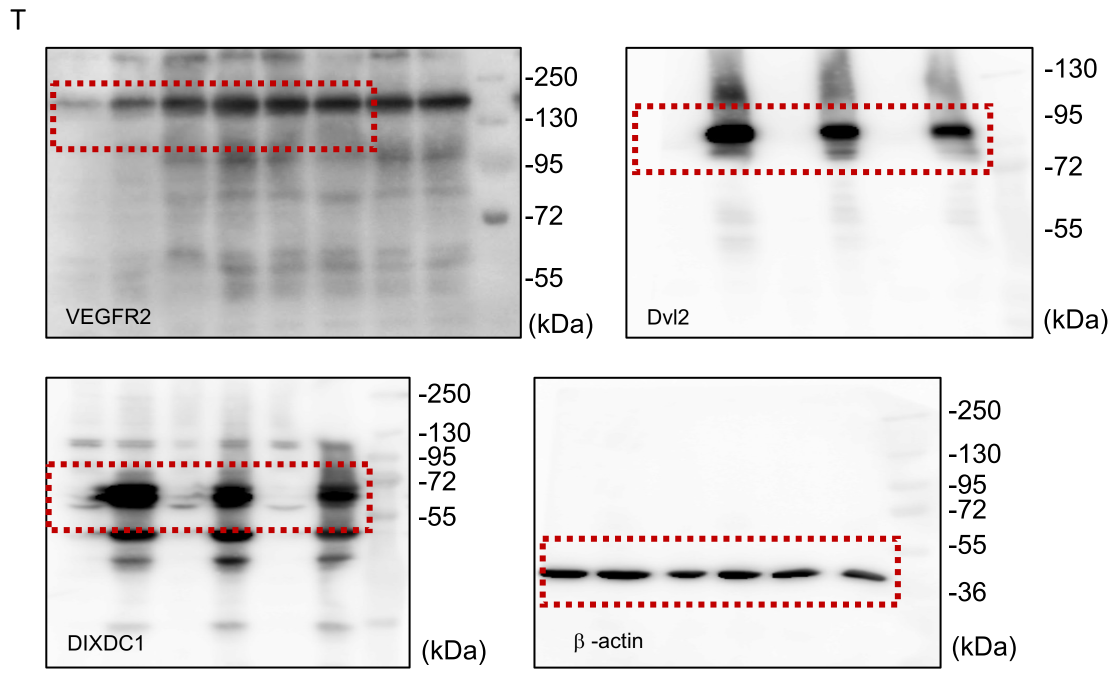
T
VEGFR2
-250
-130
-95
-72
-55
(kDa)
Dvl2
-130
-95
-72
-55
(kDa)
DIXDC1
-250
-130
-95
-72
-55
(kDa)
β -actin
-250
-130
-95
-72
-55
-36
(kDa)

Additional File 1

B

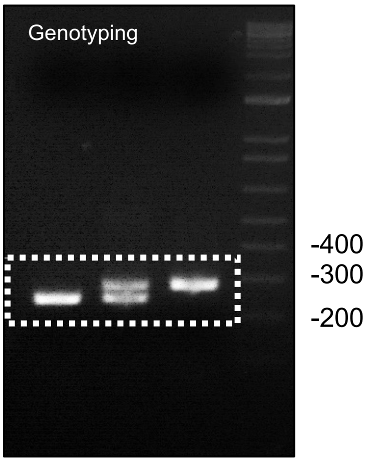

Additional File 2

G

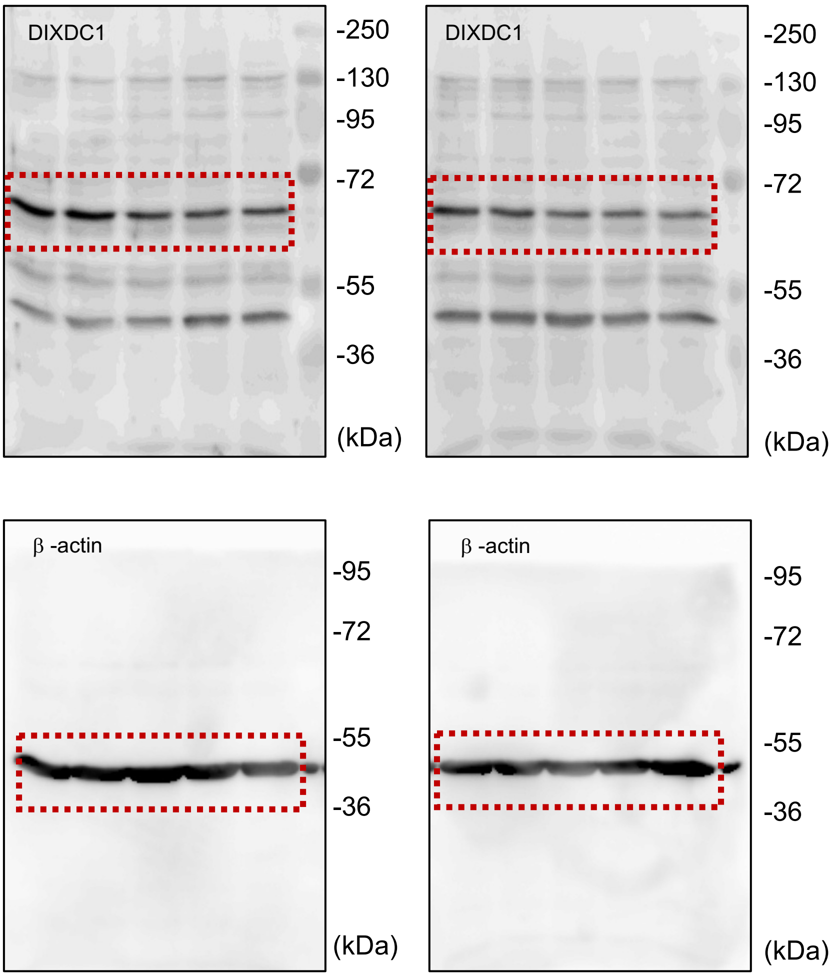

Additional File 4

A

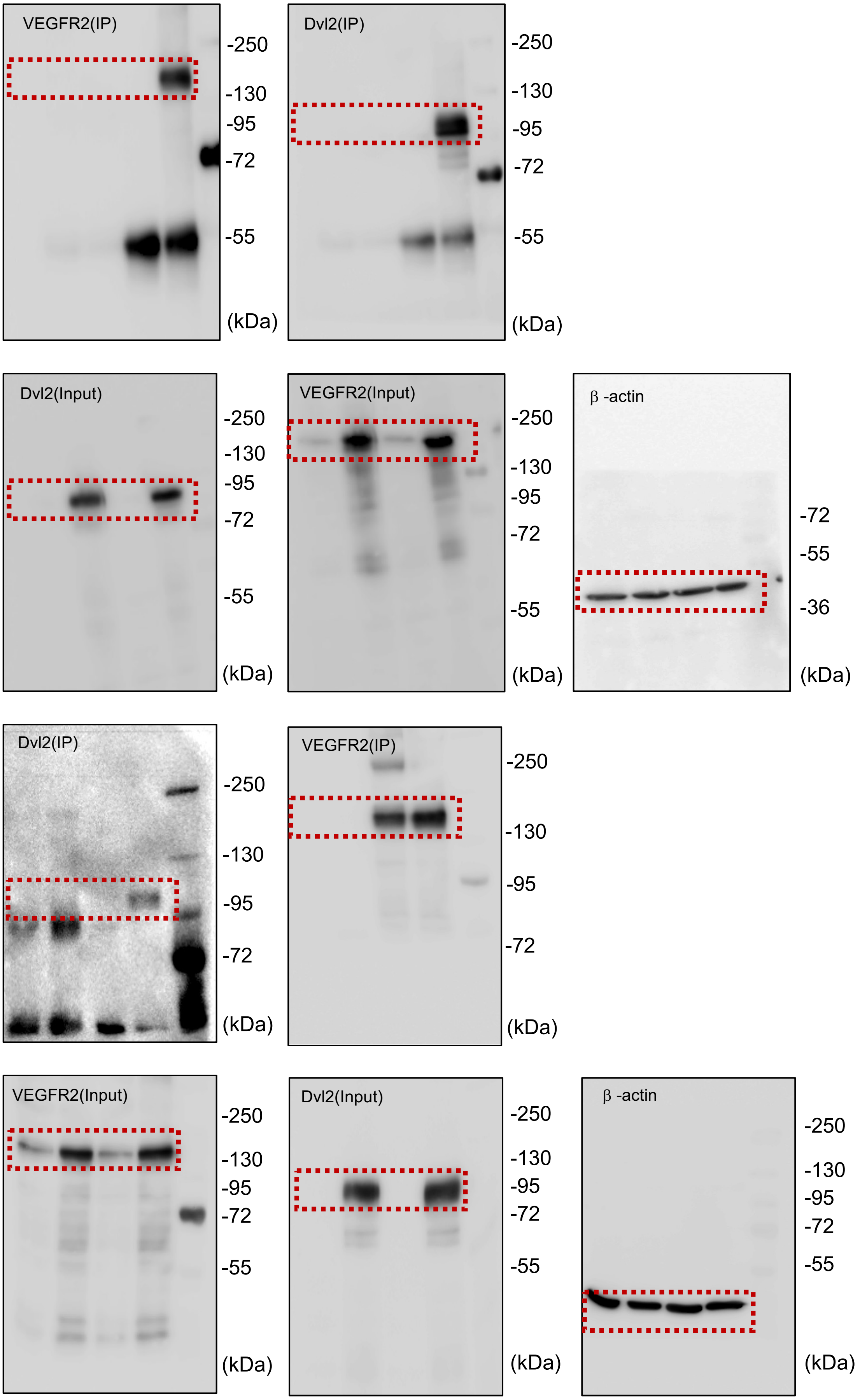

Additional File 4

B

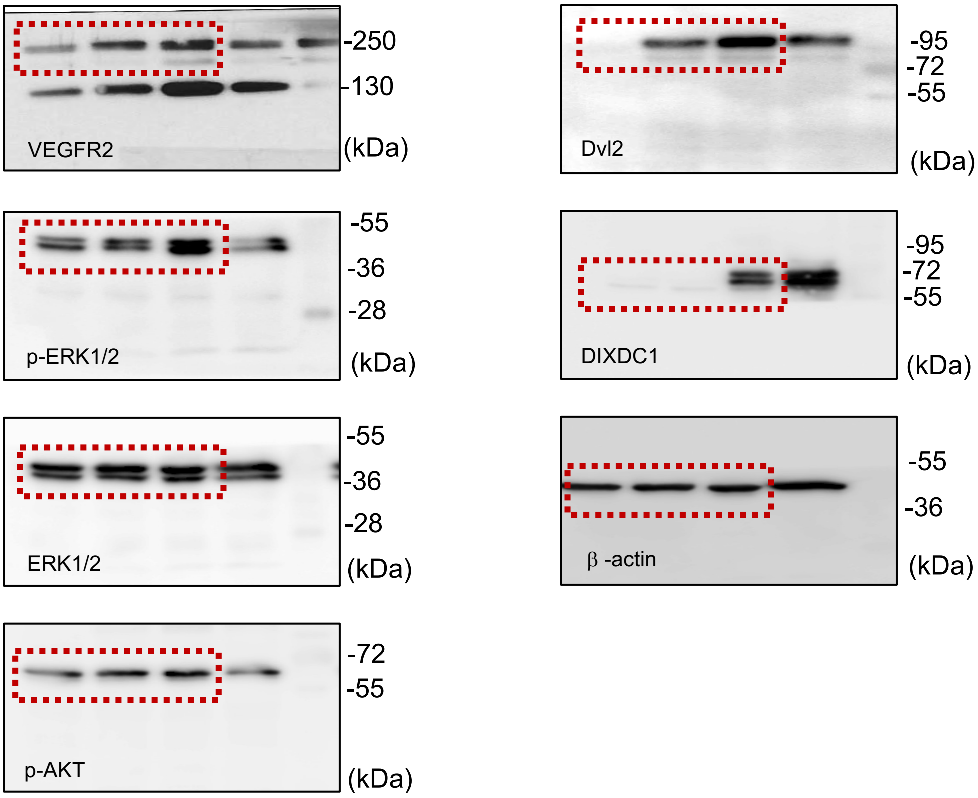

Additional File 5

A

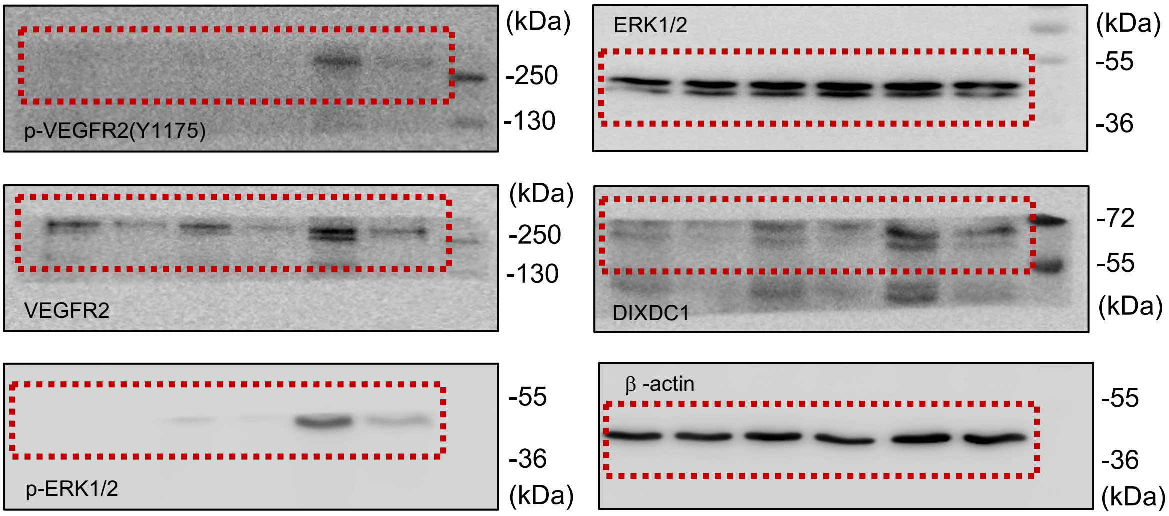

Additional File 5

F

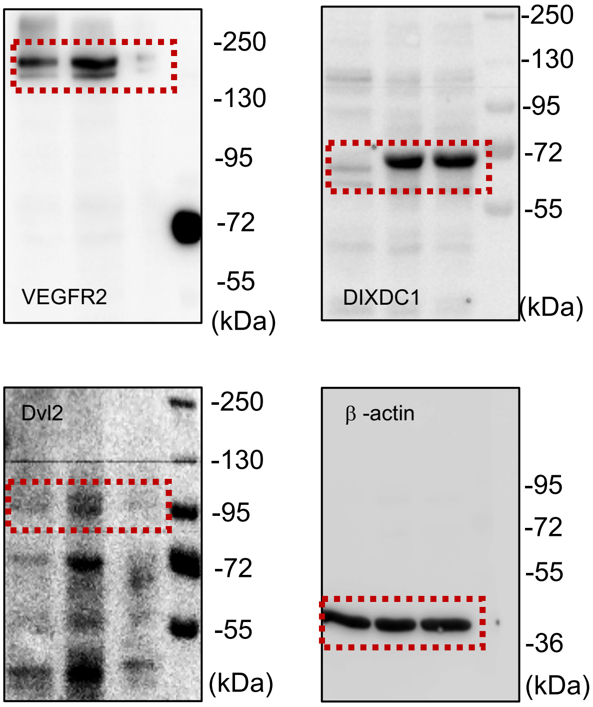

Supplement: Supplementary file 7 — Additional file 7. Uncropped Image [file 12915_2022_1240_MOESM7_ESM.pdf]
